# Lasso Proteins—Unifying Cysteine Knots and Miniproteins

**DOI:** 10.3390/polym13223988

**Published:** 2021-11-18

**Authors:** Bartosz Ambroży Greń, Pawel Dabrowski-Tumanski, Wanda Niemyska, Joanna Ida Sulkowska

**Affiliations:** 1 Centre of New Technologies, University of Warsaw, 02-097 Warsaw, Poland; b.gren@cent.uw.edu.pl (B.A.G.); p.dabrowski-tumanski@uksw.edu.pl (P.D.-T.); 2 Faculty of Physics, University of Warsaw, 02-093 Warsaw, Poland; 3 Faculty of Mathematics, Informatics and Mechanics, University of Warsaw, 02-097 Warsaw, Poland; wanda@mimuw.edu.pl

**Keywords:** topology, lasso, proteins

## Abstract

Complex lasso proteins are a recently identified class of biological compounds that are present in considerable fraction of proteins with disulfide bridges. In this work, we look at complex lasso proteins as a generalization of well-known cysteine knots and miniproteins (lasso peptides). In particular, we show that complex lasso proteins with the same crucial topological features—cysteine knots and lasso peptides—are antimicrobial proteins, which suggests that they act as a molecular plug. Based on an analysis of the stability of the lasso piercing residue, we also introduce a method to determine which lasso motif is potentially functional. Using this method, we show that the lasso motif in antimicrobial proteins, as well in that in cytokines, is functionally relevant. We also study the evolution of lasso motifs, their conservation, and the usefulness of the lasso fingerprint, which extracts all topologically non-triviality concerning covalent loops. The work is completed by the presentation of extensive statistics on complex lasso proteins to analyze, in particular, the strange propensity for “negative” piercings. We also identify 21 previously unknown complex lasso proteins with an ester and a thioester bridge.

## 1. Introduction

Unification is a key concept in which the most important properties of a given phenomena are extracted. A major goal in physics is the unification of fundamental interactions. In mathematics, common concepts are generalized in frames of category theory. In biology, homological sequences are clustered, leading to the notion of highly conserved (hence functionally important) residues. The spotted similarities spotted may guide the fundamental principles in various fields of science.

**Figure 1:**
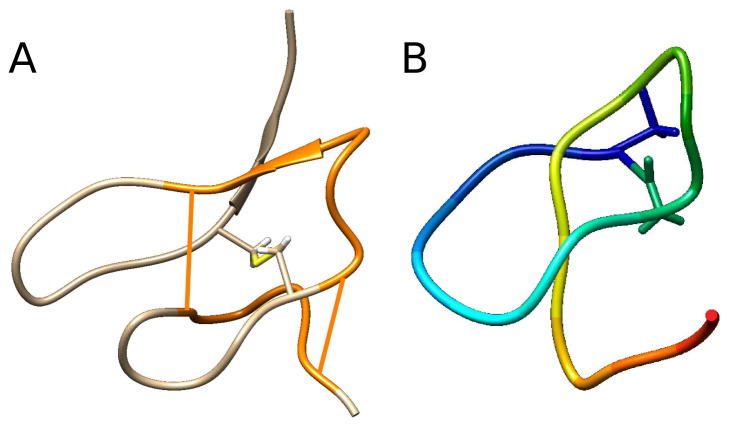
(**A**): Cysteine knot motif. The closed loop (orange) is formed by two disulfide bonds (straight stripes) and the main chain. The third cysteine bond, which pierces the closed loop, is shown explicitly. (**B**): Schematic depiction of the miniprotein Microcin J25. The loop is closed by an amide bridge (to facilitate the view, only bridge-forming residues are shown in atomic resolution).

During the last 30 years, though not expected, more entangled structures have constantly been discovered. The most well-known examples include knotted [1,2] or slipknotted [3,4] proteins, links [5,6], and chain mail structures of bacteriofag capsid [7]. In 2019, θ-curves were found [8]. In the 1990s, two other complex structures were discovered—cysteine knots [9,10,11,12,13] and lasso peptides (or lariat protoknots) [14,15,16,17,18,19]. To avoid confusion, throughout this paper, we call these miniproteins. Both types have proven therapeutic significance [11,20,21], both featuring the presence of at least one covalent loop (formed by the main chain closed by an amide or disulfide bridge), which is pierced by some portion of the chain or its bridge (see Figure 1). Moreover, some cysteine knots and miniproteins fulfill a similar function.

**Figure 2:**
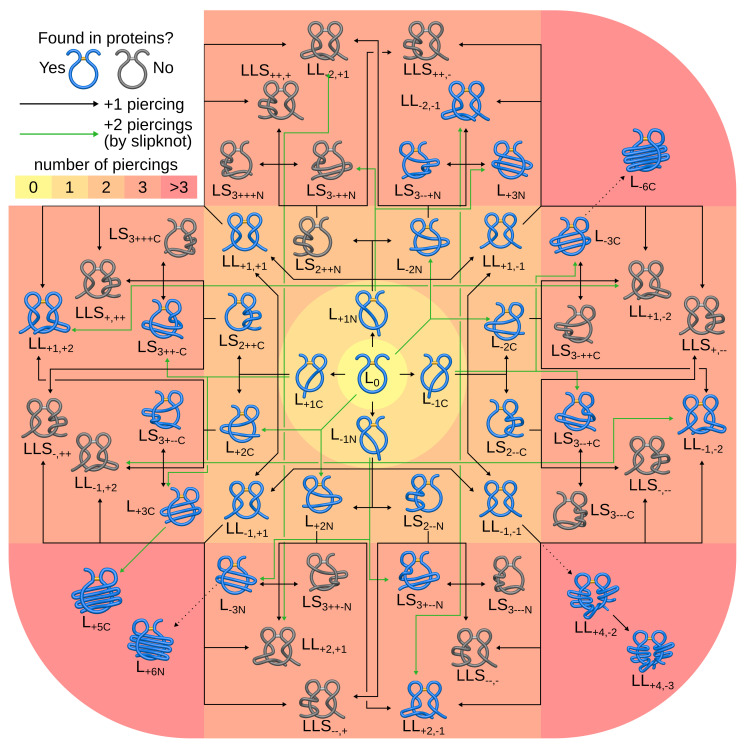
The “evolution flower” of lasso types. The figure shows all possible lasso motifs with up to three piercings and observed lassos with higher number of piercings. The structures observed in PDB are shown in blue, and those not observed (up to 3 piercings) are shown in grey. The structures are grouped according to the total number of piercings (shown by the colored background). The lasso types that can be formed by the addition or reduction of a terminal piercing are joined by a solid black line. Lasso types that can be formed by the addition or reduction of a terminal slipknot piercings are joined by a solid green line. Please note that some lasso motifs can be obtained in more than one way. The dotted lines denote the possible evolution pathway, for which however, some intermediate lasso types have not yet been identified.

**Figure 3:**
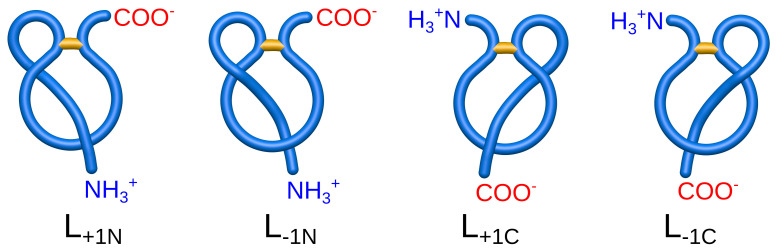
Subclasses of the $L_{1}$ major type. From left to right are $L_{+1N}$, $L_{-1N}$, $L_{+1C}$, and $L_{-1C}$.

Is this only an accident or is there any direct correspondence between the entanglement type and the fulfilled function? In 2016, a similar new entanglement type was observed—the complex lasso proteins [22,23]—in which the covalent loop closed by a disulfide (or other) bridge is pierced by one or two tails (Figure 2 and Figure 3). The complex lasso extracts the most important structural properties of cysteine knots and miniproteins, and is more general (18% of proteins with an intrachain cysteine bridge exhibit complex lasso entanglement [23]). This has forced us to think about both cysteine knots and lasso peptides as cases of the same general topological motif.

Such a viewpoint has introduced a whole new chapter to the study of complex topology proteins and has led to many new questions. For example, is it indeed the topology that determines the function? If so, are there any other functional entangled motifs? Do we know all types of complexity in proteins? Are there any non-disulfide-based complex lasso proteins other than miniproteins?

Some of those questions have been at least partially answered so far. In a previous survey [23], we reported the existence of six major classes of complex lasso proteins (with disulfide loops):

- $L_{1}$, ($L_{2}$, $L_{3}$, $L_{6}$)—with one, (two, three, six) piercing(s) of the covalent loop, where each subsequent piercing is from a different side of the loop’s surface,
- $LS_{n}$—with *n* piercings, but at least two consecutive ones from the same side of the loop’s surface (the tail winds around the covalent loop),
- $LL_{i,j}$—with *i* piercings performed by the N-terminal tail and *j* piercings by the C-terminal tail.

This classification is completed with the trivial class $L_{0}$, which gather closed loops that are not threaded (see Figure 2). A detailed description of the lasso type naming is contained in the Supplementary Materials.

The question of the lasso function may be approached with statistical analysis. However, to obtain reliable results, one has to also take into account the piercing tail (N- or C-terminal) and the piercing direction (see Figure 3). Observe, e.g., that all known miniproteins have the same piercing direction when orienting as shown in Figure 1B. If the loop begins “behind” the plane, the chain pierces always from “above” the covalent loop. Moreover, one has to be aware that one protein chain can have more then one pierced covalent loop.

To deal with those issues, we created a server and database called LassoProt to analyze complex lasso entanglement in proteins and arbitrary polymers [24]. This is available at lassoprot.cent.uw.edu.pl (accessed on 31 October 2021). This algorithm is also available in our Python package, which is called Topoly [25] topoly.cent.uw.edu.pl (accessed on 31 October 2021) and in the PyMol plug-in PyLasso [26] pylasso.cent.uw.edu.pl (accessed on 31 October 2021). Moreover, topological information is supplemented by basic biological and structural information and various filters, allowing a methodological statistical search to be conducted. Equipped with this tool, we now present a comprehensive analysis of complex lasso proteins from chemical, physical, biological, and also evolutional points of view.

Previously we conducted a statistical analysis of lassoed ideal chains (no volume, no interactions, fixed distance between neighboring atoms) [27]. We found the expression for the probability of a complex lasso formation as a function of loop and tail lengths and compared these results with the distribution of complex lassos in proteins. We found a few protein groups with short tails that have a larger probability of being complex than the ideal chains that the model provides. This means that enthalpic interactions in these proteins favor complex lasso formation. This is a strong indication that the lasso structure plays an important role in these proteins. Many of them are miniproteins or other small antimicrobial proteins with similar plug structures. Some others are enzymes, which may perform a similar function as in knots—making the active site more rigid [28].

Lasso proteins can serve as a molecular switch, as their topology depends on their oxidation potential (the first attempts towards such an application have been made so far [29]). If two stable conformations of the loop relative to the piercing chain exist, such proteins can be used as molecular machines or as a molecular memory [30], similarly to the utilization of the rotaxanes. Redox and pH-controlled lasso-based pseudorotaxanes have already been synthesized using chemical protocols [31,32]. After three decades of research, there is still keen interest in new synthesis methods for molecular rotaxanes [33,34,35,36]. Therefore, lasso peptides can provide biological inspiration for (bio)chemical design. In fact, lasso peptides were recently used as a template to obtain self-assembling protein rotaxane structures after enzymatic cleavage [37,38].

This paper is organized as follows: First we compare the results of a survey based on a non-redundant set with only the lasso types present in the entire PDB, taking into account the tail and direction of the piercing. We show that there is a strange propensity for “negative” piercings. Next, we move to the analysis of proteins comprising a few complex lassos, proteins with non-disulfide-based bridges, and structures classified, due to various reasons, as artifacts. In particular, we show that there are around 20 ester-based and thioester-based pierced loops. We also point out some gapped structures which, upon determining the whole structure, may reveal new lasso motifs.

After analyzing the structural data, we move to the biological results. We analyze the conservation of the lasso type and its function and evolution. To study the functional relevance, we introduce a method of analyzing the bulky residues and the flexibility of the chain based on the assumption that a functionally important lasso should be stabilized, as happens in the case of miniproteins. This approach allows us to show that the $L_{-1C}$ lasso type present in antimicrobial lasso proteins and the $L_{2}$ lasso type present in cytokines may be functionally relevant.

The final part of the paper is devoted to the discussion of the results, the possible utilization of complex lasso proteins, and the future research direction.

## 2. Materials and Methods

### 2.1. Protein Set

In our analysis, we used all the proteins deposited in the Protein Data Bank up to October 2020, i.e., 535,079 structures. The gaps were modeled as straight intervals. The proteins that did not pass the structure validity test described in [24] and the Supplementary Materials were counted as artifacts.

### 2.2. Lasso Type Assignment and Closed Loop Detection, Visualization

The topology assignment followed the algorithm developed in [23] with its extension introduced to deal with surface orientation [24]. The covalent loops were detected as described in [24]. Molecular graphics and analyses were performed with the UCSF Chimera package [39] and JSmol implemented in the LassoProt server [24]. Chimera was developed by the Resource for Biocomputing, Visualization, and Informatics at the University of California, San Francisco (supported by NIGMS P41-GM103311).

### 2.3. Sequence Alignment and Bridge Conservation

Sequence alignment was done using the Clustal server via its Perl web client (based on the SOAP protocol). Alignment was done for all clusters with a 40% sequential homology, for which highly redundant structures (with sequential homology over 95%) were reduced. For each alignment, the conservation of the bridge was calculated as the conservation of both bridge-forming cysteines separately. The bridge was considered highly conserved if both cysteines were present in more than 80% of the aligned structures. The bridge was considered poorly conserved if at least one cysteine was present in less than 30% of the aligned structures. In the other case, the bridge was considered medium conserved.

### 2.4. Protein Characteristics

Extracellular locations were derived from the UNIPROT database. The B-factors, mean square deviations, and locations of bulky residues were determined from the appropriate PDB file. Detailed descriptions are provided in the Supplementary Materials.

## 3. Results

### 3.1. Analysis of the Entire Protein Data Bank

#### 3.1.1. The Non-Redundant Set Is Sufficient

We analyzed all protein structures (535,079 chains in October 2020) deposited in the Proteins Data Bank, extracting those possessing a covalent loop (closed by any kind of a chemical interaction—78,723 chains). The chains were divided into topologically certain (52,832 chains) and the “artifact” class with an uncertain entanglement type (e.g., due to the gap size—25,891 chains) in accordance with to [24]. The details for the division methodology are given in the Supplementary Materials.

For each covalent loop, we determined the major lasso type (not taking the orientation and piercing tail into account) and minor lasso type (including the piercing tail and piercing orientation). The major lasso types found in the entire PDB were identical to those within the non-redundant set, except for $L{}_{5}$. All motifs present across the entire PDB were represented in the non-redundant set, except for five exceptional lasso types (see Table 1). These exceptional motifs arose from an additional or different bridge ($LL_{+1,+2}$ motif in glutaminase with PDB code 3a55 and $L_{+5C}$ motif in glucosidase with PDB code 5yj7), truncation of the piercing tail ($LL_{+4,-2}$ in the chemokine inhibitor with PDB code 1cq3) or a slight change in the position of the chain, resulting in acceptance of the piercings, which otherwise would be disregarded as too shallow ($LS_{3++-C}$ and $LS_{3--+N}$ motifs in transferrin with PDB code 1bp5).

#### 3.1.2. Piercings Tend to Arise from the Negative Side

By taking into account the piercing direction and the type of piercing tail (N-end or C-end), the major lasso type can be split into smaller (minor) subtypes. For example, the $L_{1}$ type is composed of four subtypes ($L_{-1C}$, $L_{-1N}$, $L_{+1C}$, $L_{+1N}$), with the direction of piercing described by a + or − sign and the crossing tail denoted by a capital N or C (Figure 3). For further details concerning the naming convention, see the Supplementary Materials.

The entire PDB was found to contain 35 subclasses—15 of the $L_{n}$ type, 11 of the two-sided lasso (LL) type, 8 of the supercoiling lasso type (LS), and 1 of the supercoiling two-sided lasso type (LLS). Note that not all possible lasso types were represented—in particular, there was no protein belonging to the $LS_{2++N}$ subclass, while there were examples of every other $LS_{2}$ lasso type: $LS_{2--N}$, $LS_{2++C}$ and $LS_{2--C}$. The lasso subclasses found throughout the entire PDB are shown in Figure 2, and the numbers of covalent loops for each type are contained in Table 1.

**Figure 4:**
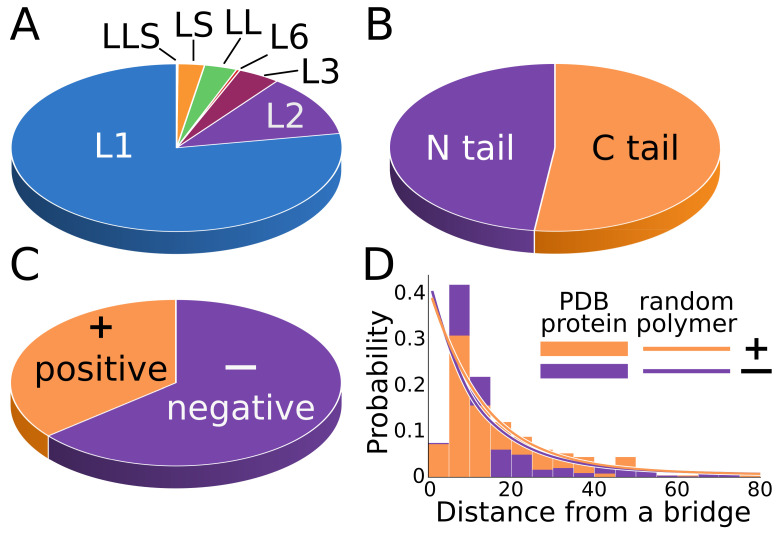
Statistics of loop-type ocurence among the non-redundant set of proteins. The pie charts present (**A**) major types, (**B**) piercing tails, and (**C**) the piercing direction. In (**B**,**C**), all lassos except the two-sided (LL) class are taken into account. (**D**) Histogram of the sequential distance between the bridge and the nearest piercing with distinction of the positive (+) and negative (−) piercings. Curves represent the theoretical prediction for a random polymer (based on the fit of the simulated data in [27]).

The number of structures in each class was found to correspond to the complexity of the structure (Figure 4A). The most popularweare singly threaded lassos. As a rule of thumb, double sided lassos and supercoiling lassos are more complex and, hence, less frequent (with LS being more frequent than LL). This trend was preserved in both the entire PDB and in the non-redundant set, showing that the latter reflects the properties of the former well.

No clear preference for the piercing tail was shown, although piercing via the C-terminus was slightly more common (52%, Figure 4B). However, there was a strong tendency for “negative” piercings. Indeed, around 64% of pierced loops in the representative structures belonged to the class with a “negative” piercing closest to the bridge (Figure 4C), taking into account all but two-sided lassos (LL). The origin of this phenomenon is not clear. A possible explanation involves the bridge chirality. In this case, the chirality effect should decrease with the increasing of the distance from the bridge. Therefore, for the “positive” piercings, the mode of distance distribution would be shifted towards larger distances compared with the “negative” piercings. Such behaviour can be observed (Figure 4D).

#### 3.1.3. Lasso Fingerprint

**Figure 5:**
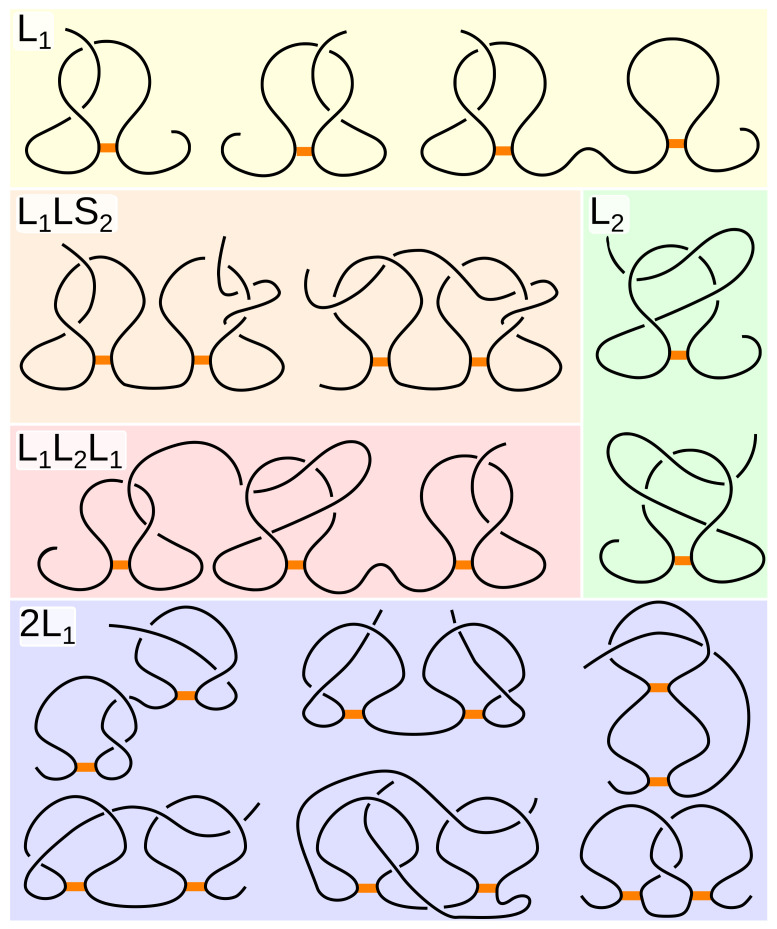
Exemplary lasso structures with fingerprints prescribed. The color boxes contain structures with the same fingerprint. The fingerprint is shown in the upper left corner of the box. The bridge is denoted with an orange strip. Note that by taking into account the piercing tail and orientation, the number of the structures in the Figure multiplies. Note also that the covalent loops in the bottom right structure are linked. Note that the structure over it has one big and one small covalent loop, both being pierced in the same place.

In some cases, the protein chain possesses more than one pierced covalent loop. A description of the global entanglement, including all covalent loops is indispensable for a proper understanding of the structural influence on the biological and mechanical chain properties. To describe the overall topology of the protein, we introduced a “lasso fingerprint” [24], a notion similar to the knot fingerprint used in the case of knotted proteins [40]. The lasso fingerprint is built as a concatenation of lasso type symbols for all pierced loops in their sequential order (with the $L_{0}$ symbols, piercing tail, and piercing direction suppressed). Such a simple and intuitive notion extracts all non-trivial parts of the protein lasso entanglement (Figure 5).

**Figure 6:**
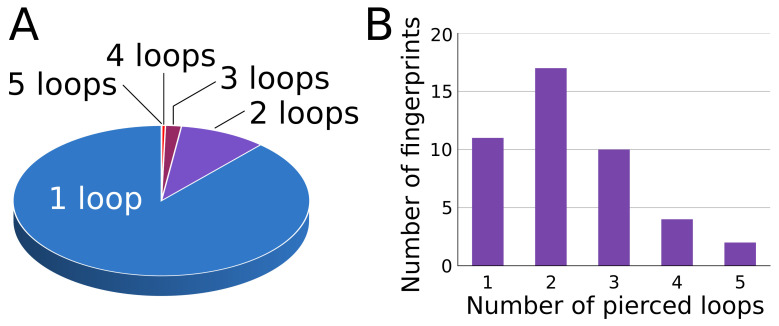
Distribution of pierced loops and lasso fingerprints in lasso proteins. (**A**) Number of pierced loops in a single chain. (**B**) Number of different lasso fingerprints depending on the number of pierced loops.

Currently, proteins with 44 different lasso fingerprints are deposited in the entire PDB database. Most structures (over 88%) posses only one pierced loop; however, ca 9% of the structures posseses two pierced loops and two structures posses 5 pierced loops (Figure 6). The greatest variety of fingerprints occurs for proteins with two pierced loops (17 different fingerprints). There are 8 different fingerprints with 6 piercings in total and 2 different fingerprints with 7 and 8 piercings (e.g., $LS_{3}\;2L_{1}\;LS_{2}$, $L_{1}\;L_{2}\;L_{1}\;2L_{2}$). Moreover, one protein possesses two loops, the first classified as $LL_{4,3}$ and the second being of the $L_{2}$ type, making 9 piercings in total.

Although the lasso fingerprint is easy to construct and contains a lot of information characterizing the chain topology (e.g., number of pierced loops, their sequential order, etc), it does not characterize the chain topology unequivocally. Observe, for example, that there are over 20 different structures that could be categorized as the $2L_{1}$ lasso fingerprint (some of them are shown in Figure 5). The development of an easy method for unequivocal multiple lasso classification could be a challenge for mathematicians but has potential importance in biology. Note that, in particular, the $2L_{1}$ fingerprint can denote the linked covalent loops (forming a Hopf link—Figure 5, bottom right panel). Such structures possessing interesting properties that were identified in [22,28].

#### 3.1.4. Non-Disulfide Bridges

**Figure 7:**
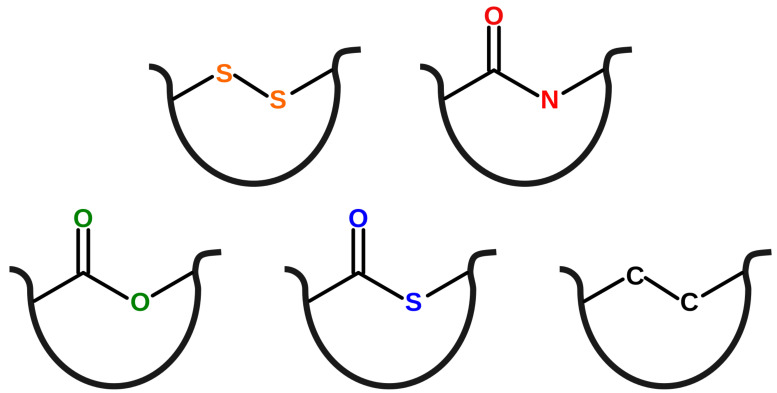
Loop-forming bonds found in PDB. From left to right, (**top** row) disulfide, amide (**bottom** row) ester, thioester, C–C. The amine, ether, and thioether bonds lack the keto-oxygen atom. The black curve denotes the protein chain.

The S-S bridge is beyond doubt the most abundant post-translational modification joining two residue side groups. Nevertheless, in some cases, the covalent loop is closed by another type of chemical bond. In the analysis of non-disulfide bonds formally present in the PDB file, one has to be very careful, as many such bonds are artificial. The details of the detection and treatment a of non-disulfide bonds is described in the Supplementary Materials. Across the entire PDB set, we identified four certain non-disulfide loop-forming bonds: N–C, C–O, C–S, and C–C (see Figure 7 and Tables S1 and S2).

Most covalent bonds between residues are a result of a post-translational enzymatic modification, although some can be an effect of an autocatalitic reaction during folding. N–C is a strong bond present in the amide linkage that is rarely found in intra-residue amines. As it is not prone to mild hydrolysis or changes in oxidation potential, it usually serves as a factor that stabilizes the structure. The C–O bond is present in ester linkages and intra-residue ether structures. The ester bond is more susceptible to hydrolysis than the amide linkage; therefore, it can serve as a kind of molecular switch between protein conformations. The C–S bond (present in thioesters and thioethers) is rather weak and can be regarded as a hidden thiol group. It can be important, e.g., in proteins with adhesive properties, which can be turned on/off depending on the conditions. The C–C bond can be formed in-between aromatic side groups, extending the system of conjugated bonds and changing the spectral properties of the molecule. Hence, it can be found in photoactive proteins. As a very stable bond under biological conditions, the C–C interaction is also introduced artificially to stabilize the structure.

Across the entire PDB, there are three groups of non-disulfide-based complex lasso proteins (i.e., with non-trivial geometry of the covalent loop):

- amide based—22 chains from X miniproteins with the $L_{-1C}$ lasso type,
- thioester based—13 chains from 10 proteins with 5 different lasso types ($L_{-1N}$, $L_{-1C}$, $L_{+2C}$, $L_{+2N}$, $LL_{+1,+2}$, $LL_{+1,-3}$),
- ester based—24 chains from 11 proteins with 7 different lasso types ($L_{-1N}$, $L_{-1C}$, $L_{+1N}$, $L_{-2N}$, $L_{+2N}$, $LL_{-1,+1}$, $LL_{+1,-1}$).

In the case of miniproteins, the amide bridge is well-known to be essential for the function (see Function section) [41]. On the other hand, there is no evidence of functional importance for the two other bridges (thioester in protein glutaminase and ester in viral protease). In particular, the thioester bridge is absent when the protein is crystallized under different conditions [42], while the C-terminus of the viral protease (forming the ester bridge in the structure with PDB code 3P06) is involved in the enzymatic reaction [43]. Identified protein structures with thioesters and ester-based loops are individual cases among all structures contained in these proteins (check the Table S2 for more details). For example, there are 120 known chain structures of M17 leucyl aminopeptidase (UniProt ID: Q8IL11), but only one of them has an ester bond. The only exception is “Myosin heavy chain, skeletal muscle, adult” (UniProt ID: P13538), which has 13 chains with an ester bridge (out of 64 chains). Hence, in these cases, the presence of the complex lasso structure is probably accidental, stemming from the conditions of crystallization.

### 3.2. Topology Conservation

In the case of known topological motifs (e.g., knots), the topology is better conserved than the sequence [40]. However, in the case of a complex lasso protein, a simple comparison of lasso fingerprints in the homology cluster can be misleading. The complex lasso motif can be introduced or removed upon mutation of only one crucial amino acid—the bridge-forming cysteine. In general, many factors contribute to the ostensible change of the topology (mutation, insufficient resolution etc), which are described in detail in the Supplementary Materials. Therefore, to study the lasso motif’s conservation, we used two complementary approaches.

The first approach followed the procedure of [44], in which the conservation of the cysteine residues (not bridges) in the homology cluster is calculated. Bridges were considered conserved if at least 80% of non-unique structures in the homology cluster had a bridge (see the Supplementary Materials for details). Structures were treated as unique if they had a similarity of at least 95%. It turned out that 405 of 734 (55%) clusters had only one unique structure. This means that, in most cases, complex lasso proteins were unique. In the remaining set of 329 clusters, the disulfide bridges were exceptionally conserved, with 72% classified as highly conserved compared with only 54% in regular proteins [44]. This high fraction also stemmed from the fact that some proteins were found to possess over 10 (all highly conserved) disulfide bridges. However, when calculating the conservation for the pierced-loop-forming bridges only, the percentage of highly conserved bridges was equal to 54%.

The second approach used to determine the lasso conservation was to search for the conservation of a lasso type among PFAM domains. The complex lasso proteins represent 275 different PFAM domains. Among the representative structures of the checked PFAM domains, we aimed to determined what was more common out of a complex lasso structures, a trivial lassos, and a structures with no disulfide bridges. It turned out that, in over 35% of cases (97 distinct PFAM domains), the complex lasso structure was conserved among most representants of the domain. On the other hand, the trivial lasso structure dominated among the representants of 24% of the distinct PFAM domains (66 cases). The reason for nonconservation of the lasso structure could be the truncation of the chain or the removal of the crucial bridge. Nevertheless, such proteins are perfect objects to study the biophysical importance of the pierced loop. The remaining 41% (112 cases) of PFAM domains most commonly had no bridge at all.

### 3.3. Complex Lasso Protein Function

The advantage of having a lasso motif is unclear. It was shown that the lasso structure can influence hormone action [45] or can serve as an on/off switch [29]. The lasso can also introduce additional stability to the protein [28]. Indeed, over 50% of the non-redundant set of lasso proteins for which a location is given in the UNIPROT database are secreted and, therefore, exposed to harsh conditions, a process that requires enhanced stability (for details see the Table S3). Moreover, a correlation was found between different lasso types and protein function [23]. In particular, most antimicrobial proteins were of the $L_{1}$ type. More specifically, the $L_{-1C}$ lasso type of antimicrobial proteins resembling the (antimicrobial) lasso peptides structurally was found to be characteristic to all families from the PFAM Defensin Clan (CL0075 Defensin/myotoxin superfamily), 4 out of 12 families from the Cystine-knot (CL0079) clan, and 4 other PFAM families containing antimicrobial proteins. This and the resemblance to miniproteins suggest that they have a common mechanism of action as molecular plugs of appropriate channels [46]. Note that we only checked families with known protein structures.

#### 3.3.1. Other Correlations

Apart from the $L_{-1C}$ lasso type, other lasso types exhibit interesting correlations. Previously, it was shown that all eucariotic proteins from the Double Psi beta barrel glucanase PFAM Clan form a Hopf-linked $L_{+1C}L_{+1N}$ lasso structure. $LS_{2--N}$ is characteristic for the PA14 superfamily, and the $L_{-2C}$ type is characteristic for the TIMP-like superfamily. The $L_{2}$ type, in general, is also found in many transport or signaling proteins, such as cytokines.

#### 3.3.2. Stabilization of the Lasso Motif as an Indirect Proof of Functionality

If the $L_{-1C}$ antimicrobial lasso proteins indeed act as molecular plugs like the miniproteins, the functional lasso motif should be stabilized by inter-molecular interactions. In the case of miniproteins, stabilization is introduced by the presence of bulky residues surrounding the piercing residue, blocking its movement. In fact, the stabilization of the lasso and, in particular, the piercing residue is indirect proof of the functionality of the motif. Indeed, for small-loop lasso proteins (for which piercing the loop is the least probable due to entropic reasons), the piercing residue is also surrounded by large residues [23].

To quantify the stabilization of the piercing residues, we used two approaches. First, we concentrated on the mechanical blocking, known from miniproteins. In this case, the piercing residue surrounded by bulky residues is located in the piece of chain with a high concentration of large-volume amino acids. We, therefore, calculated the local concentration of bulky residues and identified its local maxima. Then, we calculated the sequential distance between the piercing and the (sequentially) closest maximum (for details see the Supplementary Materials). If the piercing is stabilized mechanically, the distance should be small compared to the average.

**Figure 8:**
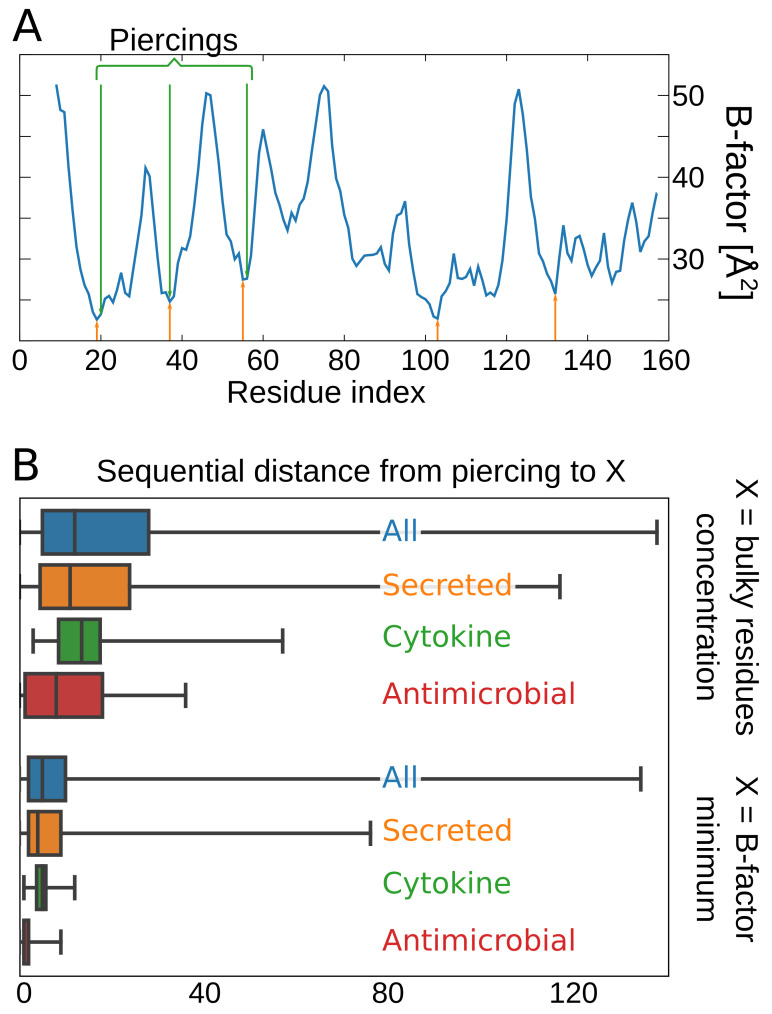
Quantifying lasso stabilization. (**A**) Comparison of the locations of piercings (green arrows) and minima of the B-factor (orange arrows) for lipocalin with PDB code 1A3Y. (**B**) Box plots of the mean distance from the local maximum of the bulky residues concentration and the mean distance from the local B-factor (or mean square deviation in the case of NMR structures) maximum. White dots represent the mean value of each protein family set. Box widths represent 1st-3rd quartile range with the horizontal line showing the 2nd quartile (median).

Secondly, the highly stable parts of a protein should be characterized by a low B-factor in the case of a crystal structure. In the case of an NMR structure, the analog of the B-factor can be the mean square deviation of positions of atoms in different models (for details, see the Supplementary Materials). We, therefore, identified the minima of the B-factor (or mean square deviation) and, similarly to the previous approach, we calculated the sequential distance between the piercing and the (sequentially) closest minimum. Again, the stabilized piercing should be characterized by a low distance compared with the average. This approach is further supported by the fact that, in many cases, the location of the piercing correlates well with the location of the calculated minimum of the B-factor [47], e.g., for lipocalin with PDB code 1A3Y (see Figure 8A). To determine the loop sizes for which the effect is still visible, we plotted both average distances (from the maximum of bulky residues concentration and minimum of B-factor)—Figure 8B. To determine the possible importance of each class, we also compared the average for all proteins with average values for secreted antimicrobial $L_{-1C}$ and cytokines with the $L_{2}$ lasso type.

It turned out that, in both approaches, the mean value corresponding to both the antimicrobial $L_{-1C}$ type and cytokines with the $L_{2}$ type was located below the mean value for all possible loop sizes. This means that, in both groups, the lasso motif is stabilized more than in an arbitrary chosen lasso structure. This implies that, in those groups, the lasso motifs can be important for the function. The mean value corresponding to the secreted proteins is closer to the mean value of the whole set, implying that, in many cases, the lasso structure may not be important for the function. and the stability of those proteins is maintained sufficiently solely by the existence of the disulfide bridges.

### 3.4. Lasso Evolution

The origin of the lasso motif and its evolution have not been studied thus far. There are two possible evolution pathways. Either the lasso covalent loop is primordial and the piercings were ”added” during its evolution by extending the piercing tail (covalent-loop-based evolution), or the fold of the protein was optimized during evolution with the formation of a disulfide bridge as the last step (fold-based evolution). In this case, all piercings would have “appeared” at the same time, upon loop closure. Possibly, different mechanisms were involved for different proteins, and establishing such a mechanism for a particular group of proteins would require an in-depth bioinformatical analysis. However, covalent-loop-based evolution seems harder to perform, so the question we asked was, are there are any traces of such evolution? In other words, could the flower in Figure 2 really be an evolution flower?

To answer this question, we searched for (evolution-based) PFAM families containing structures with different lasso types, gathered together in the same (evolution-based) PFAM clan. Co-ocurence of different lasso types in one PFAM clan is a clue that such structures could evolve from the same ancestor. As a result, we found several pairs of lasso motifs joined by membership in the same clan. All possibly evolutionarily important connections (like $L_{-1C}\to L_{-2C}$) are depicted in Figure 2. Interestingly, there 5 proteins were identified which, depending on their structures, had different topologies: $L_{1}$ or $L_{3}$ ($L_{+1C}/L_{+3C}$, $L_{-1C}/L_{-3C}$ and $L_{-1N}/L_{-3N}$). This proves that the triply pierced proteins may have evolved from singly pierced ones by adding two piercings “at once”, i.e., the slipknot conformation, known from knotted protein folding [48].

### 3.5. Possible Applications

The observation that many well known proteins carry an intrinsic topological structure opens an entirely new chapter in protein application and classification. The stability introduced by lasso proteins and probably utilized by viruses (large fraction of lasso proteins was found in many different viruses [23]) and can be also used to construct proteins with enhanced stability. Additionally, lasso proteins may form a new group of drugs or antibiotics working as the plugs of an appropriate channel, utilizing the mechanism suspected for $L_{-1C}$ type proteins. Another interesting application of (biologically optimized) lasso proteins is in nanomachinery. In general, exploring the biophysics of the lasso proteins depending on different oxidation potential of the solution may lead to many exciting discoveries.

## 4. Discussion

In this work, we have presented the results of a scrupulous analysis of all complex lasso proteins deposited in the entire RCSB PDB database. We showed that there is strong resemblance between complex lasso proteins of the $L_{-1C}$ type, lasso peptides, and antimicrobial cysteine knots. This suggests that the piercing of the covalent loop is a key structural feature of such proteins and that they can share the same mechanism of action. From this perspective, the complex lasso proteins seem to be the generalization of such a concept, with different lasso types responsible for different functions (e.g., $L_{2}$ popular among cytokines). Moreover, although lasso proteins are usually disulfide based, we also identified ester- and thioester-based lasso proteins.

The functional advantage of lasso proteins was tested based on the assumption that the functionally relevant lasso should be stabilized compared with the ”average lasso”. Following this approach, we showed that for the antimicrobial $L_{-1C}$ lasso proteins and $L_{2}$ cytokines, the lasso motif should be functionally important. On the other hand, the lasso motif may arise accidentally in secreted proteins with large numbers of disulfide bridges. Indeed, over 51% of lasso proteins are secreted, almost 36% are membrane, 15% are virial, and only 32% are intercellular proteins, according to UNIPROT. In the case of secreted proteins, there is a smaller stabilization effect, meaning that, in many secreted proteins, the lasso may not be functional.

The secretion of lasso proteins leads to the problem of lasso protein folding. The secreted proteins fold under oxidative conditions, in which the disulfide bridges form during folding. Therefore, the folding of lasso proteins may require threading of a chain through the covalent loop. This process has not been investigated in general, although some preliminary studies have been conducted [45]. This is especially interesting in the case of the supercoiling lasso, for which two threadings from the same side are required, causing folding to be especially hard to complete. The analysis of the folding problem may give clues about the problem of knotted protein folding [48]. On the other hand, knowledge about the folding process may solve the conundrum of the significant prevalence of structures with “negative” piercings. Moreover, folding simulations and experiments with truncated or elongated chains may prove the “covalent-loop-based” mechanism of lasso protein evolution, in which the more complicated lassos are thought to have evolved from less complicated ones.

The evolution of lasso proteins was studied by the comparison of different PFAM families within one PFAM clan. In particular, it seems more probable that the triply pierced lassos evolved from singly pierced rather than from doubly pierced lassos. This may explain the lack of structures with three piercings in the supercoiling manner (tail winding two times around the covalent loop, effectively piercing a loop three times from the same side), as such lasso types cannot be formed by introducing a slipknot conformation into the chain.

In this work, we also analyzed the properties of the lasso fingerprint, which describes, in the most basic way, the nontriviality of the chain. We think that this could be a valuable extension of known classification methods of proteins based on the disulfide bond pattern [49,50], enabling us to prescribe a more detailed protein functions. However, we argue that this method cannot distinguish between all possible composite lasso structures. Full classification of possible lasso motifs, taking into account all protein loops, may require a sophisticated mathematical approach, similarly to how the knot theory entered biology through knotted proteins. However, no mathematical description of lasso-like structure exists as yet.

Regarding the mathematical classification, folding process, evolution, exact function and correlation with the lasso type, free energy landscape, exact influence of the lasso on the stability and conformational ensemble of the protein, much is still unknown in the field of lasso proteins. However, on the other hand, evolutionarily optimized nanomachinery, new therapeutically significant proteins, better understanding of the influence of topology, and much more can be achieved in the future. It is worth making the effort to understand lasso proteins. They have much to offer.

### Significance

In this paper, we indicate that complex lasso proteins are a generalization of inhibitor cysteine knots and miniproteins (lasso peptides). We show that their key structural motif of loop piercing is also conserved in other antimicrobial proteins with a disulfide covalent loop. This enables us to identify the “antimicrobial lasso type”. In depth study of this phenomena is desirable from the therapeutic point of view. Moreover, we correlate another lasso type with the signaling function and introduce a technique that enables us to indicate potentially functional lasso motifs in proteins. On the other hand, the tools used by us can serve as an additional, complimentary classification method to distinguish between detailed protein functions. We also observe the puzzling piercing direction propensity. Finally, we also investigate the conservation of cysteine bridges. We show that proteins with a complex lasso topology posses exceptionally stable bridges; therefore, the non-trivial lasso motif can be also regarded as a marker of bridge stability.

## Figures and Tables

**Table 1:** Statistics of occurrences of individual loop types—numbers of loops in a given type in all PDB and in the set of non-redundant structures. Note that, in some cases, more than one pierced covalent loop may occur in a given structure (see Lasso fingerprint section).

Class	# Loops	# Repr. Loops	Class	# Loops	# Repr. Loops	Class	# Loops	# Repr. Loops
$L_{-1C}$ $L_{+1C}$ $L_{-1N}$ $L_{+1N}$ $L_{-2C}$ $L_{+2C}$ $L_{-2N}$ $L_{+2N}$ $L_{-3C}$ $L_{+3C}$ $L_{-3N}$ $L_{+3N}$ $L_{+5C}$ $L_{-6C}$ $L_{+6N}$	1459 426 2202 546 504 56 180 31 88 5 329 11 3 4 1	244 85 204 127 60 12 21 8 7 1 24 3 0 2 1	$LS_{2--C}$ $LS_{2++C}$ $LS_{2--N}$ $LS_{3--+C}$ $LS_{3+--C}$ $LS_{3++-C}$ $LS_{3--+N}$ $LS_{3+--N}$	60 95 195 4 3 1 12 9	8 4 8 2 2 0 0 3	$LL_{-1,-1}$ $LL_{-1,+1}$ $LL_{+1,-1}$ $LL_{+1,+1}$ $LL_{-1,-2}$ $LL_{-2,-1}$ $LL_{-2,+1}$ $LL_{+2,-1}$ $LL_{+1,+2}$ $LL_{+4,-2}$ $LL_{+4,-3}$ $LLS_{2--,+2}$	11 34 43 1 1 3 3 1 2 1 3 3	4 6 6 1 1 1 1 1 0 0 1 1
Sum	7845	799	Sum	379	27	Sum	106	23
Total			All loops—6330 All chains—5559			Loops in representative set—849 Chains in representative set—732		

## Data Availability

Data is contained within this article, its supplementary material and LassoProt database https://lassoprot.cent.uw.edu.pl.

## Supplementary Materials

The following are available at https://www.mdpi.com/article/10.3390/polym13223988/s1, Table S1: List of nontrivial loops closed by an amide bridge, Table S2: List of nontrivial loops closed by a thioester or ester bridge, Table S3: PFAM domain, CLAN and representative protein structure of proteins with complex lasso motifs.

## Abbreviations

The following abbreviations are used in this manuscript:

COST	European Cooperation in Science and Technology
EMBO	European Life Scientist Organisation
NIGMS	National Institute of General Medical Sciences
NMR	Nuclear Magnetic Resonance
PDB	Protein Data Bank
PFAM	Protein Families (database)
RCSB	Research Collaboratory for Structural Bioinformatics
UCSF	University of California, San Francisco
