# Supplementary material for: Lasso Proteins—Unifying Cysteine Knots and Miniproteins
Source: Polymers (Basel). 2021 Nov 18;13(22):3988. doi: 10.3390/polym13223988 (PMC8621785; doi:10.3390/polym13223988)
Supplement: Supplementary file 1 [file polymers-13-03988-s001.zip › suplement_v4.pdf]

Lasso proteins – unifying cysteine knots and miniproteins.

Supplementary Material

Bartosz A. Gren, Pawel Dabrowski-Tumanski, Wanda Niemyska and Joanna I. Sulkowska

Contents

1 Lasso type and lasso fingerprint naming **1**
1.1 Lasso naming 1
1.2 Lasso fingerprint naming 2

2 Protein set and determination of loop-forming bonds **2**

3 Conservation of lasso type **2**
3.1 Homologs with different lasso type 3
3.2 Conservation of the bridge 3

4 Quantifying stabilization of lasso motif **4**
4.1 The mean number of bulky residues 4
4.2 Localization of minima of B-factor curve 4
4.3 Calculation of minima and maxima of discrete curve 4

5 Function and location of complex lasso proteins **4**

1 Lasso type and lasso fingerprint naming

1.1 Lasso naming

There are four groups of lasso motifs, differing in the type of complexity:

- The LS group in which only one tail pierce the covalent loop, but between two consecutive piercings it winds around the loop;
- The L group in which only one tail pierce the loop and between none two consecutive piercings does it wind around the loop – the piercings are in the "there and back" maner;
- The LL group in which two tails pierce the same covalent loop (without winding around the loop);
- The LLS group in which two tails pierce the same covalent loop and at least one winds around the loop.

To each piercing one can prescribe the piercing tail (N- or C-terminal) and piercing direction. The piercing direction is determined as in [Dabrowski-Tumanski et al., 2016], utilizing the fact, that the orientation of the surface is determined unequivocally by the orientation of the chain, being the boundary of the surface. Therefore in principle, one can then describe each lasso motif by assigning for each tail a sequence of signs denoting orientation of each piercing. This method is used for LS and LLS group. For convenience, in the LS group also the total number of piercings in being attached, e.g. $LS_{2--C}, LS3_{-++}N, LLS_{--,-+}, LLS_{--,++}$. In case of LLS group the first subscript denotes the orientation of piercings for N-terminus, the second (after comma) for the C-terminus, given in the sequential order. None protein of LLS group was found in entire PDB.

In case of LL and L group all the piercings sign sequence will be alternating – after + one always gets − and *vice versa*. Therefore, for these both groups it is sufficient to give explicitely orientation only of the sequentially first piercings for both tail and the total number of piercings. In particular $L_{-2N} = L_{-+N}, L_{+3C} = L_{+-+C}, LL_{-4,+3} = LL_{-+-+,+-+}$ etc. Note, that in case of piercings performed by N-terminus, adding new piercing changes sign, e.g. adding a piercing to $L_{-2N} = L_{-+N}$ results in $L_{+-+N} = L_{+3N}$ lasso motif.

The possible lasso types for small number of piercings is shown in the Figs. 2 and 3 in main text. The number of possible lasso types rises with the total number of piercing. If the structure has n piercings in total, it can be obtained with x and y piercings performed by N- and C-terminus respectively, with $x, y \geq 0$ and $x + y = n$. There are $n + 1$ possibilities, how much piercings are performed by N-terminus (and therefore by C-terminus also). Moreover, for x piercings performed by N-terminus, each can be either positive, or negative (same for C-terminus). Therefore the number of lasso types for n piercings in total is given by $N = (n + 1) \cdot 2^x \cdot 2^y = (n + 1) \cdot 2^n$.

1.2 Lasso fingerprint naming

The lasso fingerprints are build as a concatenation of lasso motifs in their sequential order. To include only the topologically non-trivial part of the structure the L_0 loops are supressed. Moreover, to simplify the notion, the sign and the piercing tail are also supressed. Moreover, the symbols denoting the same motif present sequentially one after another are merged, e.g. instead of L_1L_1 we write $2L_1$.

2 Protein set and determination of loop-forming bonds

In the analysis we used all the protein chains deposited in RCSB database, found in October 2020 – 535,079. 78,723 (14.7%) chains possessed a linkage between residues (either disulfide, or N-C, C-O, C-C or C-S). 52,832 (67.1%) out of those chains were topologically certain, i.e. possessed either no gap, or the gap was less or equal to 6 residues. Moreover, the $C\alpha$ atoms of consecutive residues were located in resonable distance (2.0-4.2 Å). The remaining 32.9% of chains were classified as artifacts. Both sets were analyzed independently using algorithm described in [Niemyska et al., 2016, Dabrowski-Tumanski et al., 2016]. Finally, 8,527 chains topologically certain complex lasso proteins were identified. Note, that these proteins constitute 16.1% fraction of all topologically certain protein chains with any kind of intra-residue linkages.

18 complex lasso proteins were build on non-disulfide-based covalent loop (described in main text). In general, the information about the non-disulfide bridge is extracted from the LINK line in the PDB file. This however has to be filtered scrupulously, as in many cases such information is misleading. The possibly artificial LINKs in PDB file include:

- To high energy of the bond (e.g. O-O bond);
- Wrong number of substituents (e.g. 7 substituents for carbon atom, with only 4 possible);
- Wrong geometry of bonds (e.g. too small or to large angle between substituents)
- Wrong bond distance (e.g. 10 Å);
- Mixture of positions of atoms from alternative structures.

In some cases there is no sharp criterion to distinguishing between real and artificial bond. In such cases one can compare the structure with its homolog. The formation of nonstandard bond joining residues involves rather complex cell mechanisms, therefore it is supposed to be functionally important for the protein. Hence such bonds should be also present in homological chains with the same function.

The complex lasso proteins with non-disulfide-based pierced covalent loop are summarized in Tables S1 and S2:

Table S1: List of nontrivial loops closed by an amide bridge. In the parenthesis the indices of loop-forming residues are given.

Lasso topology	Loops
L_{-1C}	$1RPB_A$ (1-9); $1RPC_A$ (1-9); $2LS1_A$ (1-9); $2LTI_A$ (1-9); $2LX6_A$ (1-8); $2M37_A$ (1-9); $2M8F_A$ (1-9); $2MFV_A$ (1-7); $2MW3_A$ (1-9); $2N5C_A$ (8-1); $2N6V_A$ (1-9); $3NJW_A$ (1-9); $4NAG_A$ (1-7); $4NAG_B$ (1-7); $5JQF_A$ (1-9);

3 Conservation of lasso type

The lasso type is prone to change upon mutation of only one but crucial residue – the bridge forming cysteine. Moreover, there are several reasons for which the bridge can be missing in the crystal structure, hidering the lasso conservation analysis:

- lack of bridge due to insufficient structure resolution;
- addition of artificial bridge due to misinterpretation of the electron density;
- lack of bridge due to its damage caused by long exposure to radiation.

Furthermore, the lasso motif may change among homology cluster, if the piercing is close to the termini or to the bridge – the terminus may be truncated destabilizing or reducing the piercings, while slight movement of the chain may shift piercing closer towards the bridge, causing piercing reduction (the arbitrary criterion is that the piercing must be located at least 3 residues from the bridge). This altogether hiders analysis of the conservation of lasso motif in the homology cluster. The examples of different reasons for change of lasso type are given in the next subsection.

Table S2: List of nontrivial loops closed by a thioester or ester bridge. In the parenthesis the indices of loop-forming residues are given.

Bond type	Uniprot ID	Structures with lasso	# all structures	% structures with lasso	Lasso types
thioester C–S	D9IEJ2	4ZYP_C (69-212);	8	12.5	$LL_{+1,+2}$
	P19491	4P9H_G (65-115);	169	1,2	L_{-1N}
	P23793	5VHY_B (718-773); 5VHY_D (718-773);	4	50	L_{-1N}
	Q0ED31	1S9R_A (398-950); 1S9R_B (398-951);	27	3.7	$LL_{+1,-3}$
	Q1WDM0	5DUM_A (90-135);	10	10	L_{-1C}
	Q6TS43	5H0S_C (299-1265);	22	4.5	L_{+2C}
	Q94218	5XDA_E (461-503);	8	12.5	L_{-1C}
	Q99972	4WXQ_A (245-433);	13	7.7	L_{-1C}
	Q9AQQ8	3A55_A (47-156); 3A55_B (47-156);	8	25	$LL_{+1,+2}$
	Q9BJF5	4YGA_H (51-104)	45	2.2	L_{-1N}
ester C–O	A0A0D0F5I0	5WTI_Z (291-837)	1	100,0	$LL_{-1,+1}$
	A0A1L8F5J9	5TQ2_B (44-266)	60	1.7	L_{+1N}
	D5AHU8	5DBU_G (6-195)	23	4.3	L_{+1C}
	O00308	5TJQ_A (500-664)	7	14	L_{+1C}
	P00336	5LDH_A (188-333); 5LDH_B (188-333)	8	25	L_{-1N}
	P31539	5VYA_D (213-387);	61	1.6	L_{+1N}
	P97438	6CQ9_A (37-268);	42	2.4	L_{+1C}
	Q1RS72	5ERB_C (139-614);	1	100	L_{-2N}
	Q2PBR5	3P06_A (738-830);	1	100	L_{+1N}
	Q8IL11	4X2T_G (512-603);	120	0.83	L_{+1N}
	P13538	1M8Q_D (506-764); 1MVW_A (502-764,505-762); 1MVW_M (502-764,505-762) 1O18_D (506-764); 1O19_A (505-762,502-764); 1O1A_A (505-762); 1O1A_D (506-764); 1O1C_A (502-764,505-762); 1O1C_D (506-764,506-764); 1O1E_A (502-764,505-762); 1O1E_D (506-764); 1O1G_A (502-764,505-762); 1O1G_D (506-764);	64	20	L_{+1N}; L_{+2N}; $LL_{+1,-1}$

3.1 Homologs with different lasso type

Lack of bridge Chain A of human hydroxylase with PDB code 4GWM has no disulfide bridge compared to its 100% homolog – chain B. The reason is probably a long exposure to radiation [Arolas et al., 2012].

Doubtful bridge Transport protein with PDB code 2WWX is the solely one structure with complex topology among 688 proteins in the homology cluster. Moreover, almost 99% of its homologs (671 structures) has no disulfide bridge at all. Another example can be oxidoreductase with PDB code 2YAU exhibiting L_{+2N} topology – none of is homologs (including five 100% sequential homologs) have any threaded loop.

Piercing to close to terminus Proteins with $LL_{+4,-2}$ and $LL_{+4,-3}$ lasso types (PDB codes 1CQ3, 2FFK, 2FIN, 2GRK) are in the same homology cluster. However the structure with $LL_{+4,-2}$ lasso type posses also the third crossing performed by C-terminus. This crossing is however too close to the terminus (2 residues) and therefore was counted as inessential. On the other hand, in case of $LL_{+4,-3}$ topology proteins the last crossing is placed in the distance of 3 residues from the terminus, therefore it was included in our analysis.

Piercing to close to the bridge Membrane protein with PDB code 3G5C has two sequentially identical chains (being the only representants of homology cluster). These chains however differ in topology, as chain A has no pierced covalent loop, whereas chain B has one closed loop with L_{-1C} lasso type. The difference stems from the fact, that in chain A the piercing is located 2 residues from the closed loop (hence it is reduced), while in chain B the distance is 3 residues (and it is preserved).

3.2 Conservation of the bridge

To avoid the aforementioned problems in investigated the entanglement conservation we followed the procedure described in [Thangudu et al., 2008]. First, in each homology cluster of 30% sequential homology we reduced highly homological sequences (with sequential homology larger than 95%). In 65% of (reduced) clusters only one sequence was contained. That means, that in most cases complex lasso proteins are unique.

In the rest of the clusters, containing at least 2 sequentially different chains, we calculated the conservation of the bridge. The structures were aligned using Clustal server via its Perl web client. The cysteine residue was called conserved between two seqences, if its index was shifted maximally by one. The bridge was called highly conserved if both bridge-forming residues were conserved in at least 80% of sequences, poorly conserved if at least one residue was conserved in less than 30%. In other case the bridge was

called medium conserved. Note, that in case of clusters containing less than 5 sequences, the bride was called highly conserved if the bridge-forming cysteines were conserved in all the seqences.

The other method used by us to determine the lasso conservation was to prescribe to each lasso the PFAM domain it belong to. Then we obtained the list of lasso types for each PFAM domain. Next we calculated the fraction of complex lasso, trivial lasso and no-loop structures.

4 Quantifying stabilization of lasso motif

To quantify the stabilization of lasso motif we calculated the sequential distance between piercing and the minimum of B-factor (or mean square fluctuation) and the maximum concentration of the bulky residues. These methods are described in subsections below.

4.1 The mean number of bulky residues

To calculate the steric hindernance in vicinity of lasso piercing we calculated, how many bulky residues are in the sequential distance of 2 residues in each direction from the piercing (i.e. how many bulky residues are among 5 residues, middle one being the piercing residue). As the bulky residues we considered those with average volume $\geq 150\dot{A}^3$, determined for the burried-ligand-water set in [Tsai et al., 1999], i.e. Arg, Trp, Met, Ile, Tyr, Leu, Phe, His. Therefore we obtained a curve for which for each residue index a natural number $\in 1 \dots 5$. For such curve we determined its maxima (according to the procedure described in following subsection) – the maxima of the bulky residue concentration. Finally, for each piercing we calculated the sequential distance from the nearest (in terms of sequential distance) maximum of bulky residues concentration.

4.2 Localization of minima of B-factor curve

To measure the general stability of each residue, the B-factor of $C\alpha$ atom for crystal structure or mean square displacement of $C\alpha$ atoms for NMR structures was used. The mean square displacement was calculated from the first model. This resulted with a specific value for an residue index. Than we calculated the minima of such curve (as in the subsection below) obtaining the minima of B-factor. Next, for each piercing the lowest sequential distance from the B-factor (or mean square deviation) was calculated.

4.3 Calculation of minima and maxima of discrete curve

The minima and the maxima of the discrete curves were calculated with similar algorithms, with the exception, that the values of 4 and 5 in case of the concentration of bulky residues were classified automatically as maximum.

The procedure of finding an extrema starts with smoothing the curve with the running average in the window of 1/10 size of the protein (the floor function – the largest integer value lower or equal to 1/10 of the size). Than the local minima (maxima), i.e. the points for which both neighbouring values are greater (lower) than the value in that specific points are taken. Next we "restored" the minima (maxima) of the original curve in the vicinity of the local minima (maxima) found for the smoothed curve. In the window equal to 1/5 of the protein size centered in the minimum (maximum) of the smoothed curve we searched for the minimal (maximal) value. This value was called minimum (maximum) of the analyzed discrete curve.

5 Function and location of complex lasso proteins

For each PFAM domain we checked what lasso motifs represent such domain. Moreover, for each domain we extracted the representative structure from the non-redundant set, the PFAM clan and cellular location they represent (C – cellular, M – membrane, S – secreted). For the representative protein we also checked the lasso loop size. The data are included in Tab. S3.

Table S3: The table containing the information about PFAM domain, CLAN and representative protein chain. The different lasso motifs found within PFAM domain are placed in the "Lasso types" column seperated with semicolon. Representative protein chain always has the first of aformentioned lasso types. Abbreviations used in "Cellular location" column: C – cellular, M – membrane, S – secreted.

**PFAM identifier**	**PFAM domain**	**PFAM clan**	**Lasso types**	**Representant**	**Lasso loop size**	**Cellular location**
PF00007	Cystine-knot domain	CL0079	L_{-1C}	$5BPU_A$	56	MS
PF00012	Hsp70 protein	CL0108	L_{-1N}	$4B9Q_A$	483	CM
PF00014	Kunitz/Bovine pancreatic trypsin inhibitor domain	–	L_{-1N}; L_{+1N}; L_{-1N},L_{-1N}	$5M4V_A$	22	–
PF00020	TNFR/NGFR cysteine-rich region	CL0607	L_{-1N}; L_{-2C}	$3ON9_A$	138	–
PF00024	PAN domain	CL0168	L_{-1C}; L_{-1N}	$1KI0_A$	129	S
PF00026	Eukaryotic aspartyl protease	CL0129	L_{-1N}	$1SGZ_A$	166	CM
PF00033	Cytochrome b/b6/petB	CL0328	L_{-1C}	$2FYN_B$	25	–
PF00040	Fibronectin type II domain	CL0602	L_{-1N}	$1YC0_I$	22	S

Continued on the next page

Table S3 – continued from previous page

PFAM identifier	PFAM domain	PFAM clan	Lasso types	Representant	Lasso loop size	Cellular location
PF00047	Immunoglobulin domain	CL0011	L_{-1C},L_{-1N}; L_{-1N}	3J70$_D$	36,68	–
PF00048	Small cytokines (intecrine/chemokine), interleukin-8 like	–	L_{-2C}; L_{+1C}; L_{-2C},L_{+1C}; L_{-2C},L_{-2C}; L_{+2C}; L_{+2C},L_{+2C}	4HCS$_A$	26	M
PF00051	Kringle domain	CL0602	L_{-1C}; L_{-1N}	6H8M$_A$	65	S
PF00054	Laminin G domain	CL0004	L_{-1N}	1KDK$_A$	25	S
PF00055	Laminin N-terminal (Domain VI)	CL0202	L_{-1C}; LS_{2--C},L_{-1N},L_{-1N}; L_{-1N}; LS_{2--C},L_{-1N}	4OVE$_A$	34	CS
PF00057	Low-density lipoprotein receptor domain class A	–	L_{-3N}; L_{-1N}	2RD7$_C$	93	MS
PF00061	Lipocalin / cytosolic fatty-acid binding protein family	CL0116	L_{-3N}; L_{-1N}; L_{-1N},L_{-1N}; L_{-3N},L_{-3N}; L_{-2N}	2L5P$_A$	106	S
PF00062	C-type lysozyme/alpha-lactalbumin family	CL0037	LS_{3+--N}	2FYD$_B$	90	S
PF00064	Neuraminidase	CL0434	L_{-1C}; L_{+2C}	4MWJ$_A$	327	–
PF00068	Phospholipase A2	CL0629	L_{-1N}	3UX7$_A$	74	–
PF00069	Protein kinase domain	CL0016	L_{-1N}	4Y8D$_C$	53	CMS
PF00071	Ras family	CL0023	L_{+1N}	2WWX$_A$	101	CM
PF00074	Pancreatic ribonuclease	–	L_{+2C}	2LT5$_A$	76	C
PF00080	Copper/zinc superoxide dismutase (SODC)	–	L_{-1N}; L_{-1N},L_{-1N}	4RVP$_A$	90	CMS
PF00082	Subtilase family	–	L_{+1N}; L_{+1C}; LS_{3++-C}; $LL_{+1,-1}$; L_{-1N}	5YL7$_A$	48	–
PF00083	Sugar (and other) transporter	CL0015	L_{+1N}	6H7D$_A$	373	M
PF00084	Sushi repeat (SCR repeat)	CL0001	L_{-1N}	1C1Z$_A$	39	–
PF00085	Thioredoxin	CL0172	L_{-1N}	2IWT$_B$	48	CS
PF00088	Trefoil (P-type) domain	CL0630	L_{-1C},L_{-1C}; L_{-1C}	1PCP$_A$	28,27	S
PF00089	Trypsin	CL0124	L_{-1N}; L_{-1C},L_{-1N}	2KAI$_I$	22	–
PF00092	von Willebrand factor type A domain	CL0128	L_{-1N}; L_{+1N}; L_{+1C},L_{-2C}	1KNT$_A$	22	–
PF00093	von Willebrand factor type C domain	CL0451	L_{-1N}	1Z78$_A$	62	CS
PF00094	von Willebrand factor type D domain	–	L_{-1C}; L_{-1N}	4NT5$_A$	50	S
PF00095	WAP-type (Whey Acidic Protein) 'four-disulfide core'	–	L_{-1C},L_{-1C}; L_{-1C}; L_{-1N}	1UDK$_A$	31,22	S
PF00100	Zona pellucida-like domain	CL0159	L_{-1N}	3QW9$_A$	70	MS
PF00102	Protein-tyrosine phosphatase	CL0031	L_{+1N}	5K24$_A$	56	CM
PF00112	Papain family cysteine protease	CL0125	L_{-1N}; L_{-1C}	4YYQ$_A$	52	–
PF00124	Photosynthetic reaction centre protein	–	L_{-1C}	3KZI$_O$	26	CM
PF00127	Copper binding proteins, plastocyanin/azurin family	CL0026	L_{-2C},L_{-1C}	1MDA$_M$	33,31	M
PF00128	Alpha amylase, catalytic domain	CL0058	L_{+1N}; L_{-1N}; L_{-1C}	3VM5$_A$	46	–
PF00129	Class I Histocompatibility antigen, domains alpha 1 and 2	CL0343	L_{-1N}; L_{+1C}	4HKJ$_D$	150	M
PF00141	Peroxidase	CL0617	L_{-2N}	1H5A$_A$	205	CS
PF00143	Interferon alpha/beta domain	CL0053	L_{+1C}	3PIW$_A$	96	S
PF00149	Calcineurin-like phosphoesterase	CL0163	L_{+2N}; L_{+1N}; L_{-1N}	1OI8$_A$	335	M
PF00150	Cellulase (glycosyl hydrolase family 5)	CL0058	L_{+1N}; L_{+1C}	5Y6T$_A$	67	CS
PF00160	Cyclophilin type peptidyl-prolyl cis-trans isomerase/CLD	CL0475	L_{-2N}	5EX1$_A$	124	–
PF00161	Ribosome inactivating protein	–	L_{-1N}; L_{+1C}	4LGP$_B$	51	CMS
PF00168	C2 domain	CL0154	L_{+1C}	6EI6$_B$	114	C
PF00188	Cysteine-rich secretory protein family	CL0659	L_{-1C}; L_{-1C},L_{-1C}	6ANY$_A$	89	–
PF00194	Eukaryotic-type carbonic anhydrase	–	LS_{2++C}; L_{+1C}	4YGF$_A$	151	–
PF00197	Trypsin and protease inhibitor	CL0066	L_{-1N}	6KV2$_B$	45	–
PF00209	Sodium:neurotransmitter symporter family	CL0062	L_{-1C}	6M17$_E$	54	CMS
PF00232	Glycosyl hydrolase family 1	CL0058	L_{+5C}; L_{+1C},L_{+1C}; L_{+1C}	5YJ7$_A$	255	–
PF00234	Protease inhibitor/seed storage/LTP family	CL0482	L_{+1N}	1B1U$_A$	57	CS
PF00236	Glycoprotein hormone	CL0079	L_{-1C},L_{-1N}	1FL7$_B$	50,85	–
PF00246	Zinc carboxypeptidase	CL0035	L_{+1N}; L_{+1C}	3MN8$_C$	41	–
PF00255	Glutathione peroxidase	CL0172	L_{+1N}	2RM5$_A$	49	–
PF00264	Common central domain of tyrosinase	CL0205	L_{-2C}; L_{-2C},L_{+2C}; L_{-1N}	4Z0Y$_A$	64	C
PF00274	Fructose-bisphosphate aldolase class-I	CL0035	L_{-1N}	5O0W$_E$	68	CMS
PF00280	Potato inhibitor I family	CL0367	L_{+1C}	1TIN$_A$	46	–
PF00288	GHMP kinases N terminal domain	CL0329	L_{-2N}	1KKH$_A$	175	C
PF00324	Amino acid permease	CL0062	L_{+1N}	6KKR$_A$	464	M

Continued on the next page

Table S3 – continued from previous page

PFAM identifier	PFAM domain	PFAM clan	Lasso types	Representant	Lasso loop size	Cellular location
PF00326	Prolyl oligopeptidase family	CL0028	L_{-1C}	5TXE$_D$	9	CS
PF00330	Aconitase family (aconitate hydratase)	–	L_{+1C}	4KP1$_A$	264	C
PF00332	Glycosyl hydrolases family 17	CL0058	L_{-1N}	2JON$_A$	47	S
PF00340	Interleukin-1 / 18	CL0066	L_{-2C}	1IRA$_Y$	44	CMS
PF00370	FGGY family of carbohydrate kinases, N-terminal domain	CL0108	L_{+1N}	3KZB$_A$	39	–
PF00400	WD domain, G-beta repeat	CL0186	L_{-1C}	5GSA$_B$	324	C
PF00403	Heavy-metal-associated domain	CL0704	L_{-1N}	1DO5$_A$	87	CMS
PF00405	Transferrin	CL0177	L_{+1N},L_{-1N},LS_{2--N}; LS_{2--N}; LS_{3--+N}; LS_{3--+N},L_{-1N},LS_{2--N}; LS_{2--N},L_{-1N},LS_{2--N}; LS_{2--N},L_{+1N},L_{-1N},LS_{2--N}; L_{-1N},LS_{2--N}; LS_{3--+N},L_{+1N},L_{-1N},LS_{2--N}; L_{-1N},LS_{3--+N}	1BIY$_A$	195,280, 223	CMS
PF00419	Fimbrial protein	CL0204	L_{-1N}	5CYL$_D$	173	CM
PF00423	Haemagglutinin-neuraminidase	CL0434	L_{-1N}	6P7S$_B$	65	M
PF00431	CUB domain	CL0164	L_{-1C}; L_{-2C},L_{-2C}	1Q3X$_A$	119	S
PF00445	Ribonuclease T2 family	–	L_{-1C},L_{-1C}; L_{-1C}	1SGL$_A$	59,34	C
PF00450	Serine carboxypeptidase	CL0028	L_{+1N}	1AC5$_A$	267	CM
PF00457	Glycosyl hydrolases family 11	CL0004	L_{-1C}	3WP6$_A$	169	S
PF00464	Serine hydroxymethyltransferase	CL0061	$LL_{-1,-1}$; $LL_{-2,-1}$	4PFF$_A$	240	–
PF00483	Nucleotidyl transferase	CL0110	L_{+1N}	4JD0$_A$	210	C
PF00486	Transcriptional regulatory protein, C terminal	CL0123	L_{+1N}	3LY7$_A$	65	M
PF00496	Bacterial extracellular solute-binding proteins, family 5 Middle	CL0177	L_{+1C}	2D5W$_A$	145	M
PF00497	Bacterial extracellular solute-binding proteins, family 3	CL0177	L_{+3C}	6H1U$_A$	102	MS
PF00503	G-protein alpha subunit	CL0023	L_{-2C}	6LFM$_D$	28	–
PF00509	Haemagglutinin	–	L_{-1C}	6II4$_A$	43	–
PF00523	Fusion glycoprotein F0	CL0595	L_{+1N}; L_{-1C}	5EJB$_B$	122	–
PF00530	Scavenger receptor cysteine-rich domain	CL0550	L_{-1C}; L_{-1C},L_{-3C}	2JOP$_A$	66	M
PF00535	Glycosyl transferase family 2	CL0110	L_{+3N}; L_{+2N}	6S24$_A$	82	C
PF00540	gag gene protein p17 (matrix protein)	CL0074	L_{+1C}	4DQE$_A$	23	–
PF00544	Pectate lyase	CL0268	L_{-1N}	1QCX$_A$	135	S
PF00545	ribonuclease	–	L_{-3C}	3AGN$_A$	54	C
PF00553	Cellulose binding domain	CL0203	L_{+1N}; $LL_{+1,-1}$	2CKR$_B$	241	–
PF00557	Metallopeptidase family M24	–	$LL_{-1,+1}$	1R58$_A$	221	C
PF00561	alpha/beta hydrolase fold	CL0028	L_{+1C}	4HS9$_A$	58	S
PF00576	HIUase/Transthyretin family	CL0287	L_{-3N}	1QAB$_E$	105	CS
PF00578	AhpC/TSA family	CL0172	L_{+1N}	2CVB$_A$	129	–
PF00594	Vitamin K-dependent carboxylation/gamma-carboxyglutamic (GLA) domain	CL0001	L_{-1N}; L_{-1N},L_{-1N}	1BTH$_Q$	22	–
PF00599	Influenza Matrix protein (M2)	–	L_{-1N}	6S0Y$_A$	57	CMS
PF00625	Guanylate kinase	CL0023	L_{+1N}	1S4Q$_A$	154	C
PF00652	Ricin-type beta-trefoil lectin domain	CL0066	L_{+2C}	3WMY$_A$	269	S
PF00664	ABC transporter transmembrane region	CL0241	L_{-1N}	6QV2$_E$	60	CMS
PF00683	TB domain	–	L_{-1N}	1KSQ$_A$	26	S
PF00688	TGF-beta propeptide	CL0055	L_{-1N}	3B4V$_D$	45	S
PF00704	Glycosyl hydrolases family 18	CL0058	$LL_{+1,-1}$; L_{+1N}	5XWQ$_A$	158	–
PF00706	Anenome neurotoxin	CL0075	L_{-1C}	1AHL$_A$	31	S
PF00711	Beta defensin	CL0075	L_{-1C}	1E4Q$_A$	16	S
PF00726	Interleukin 10	CL0053	L_{+1C}	4DOH$_A$	94	S
PF00732	GMC oxidoreductase	CL0063	L_{+1N}	1CF3$_A$	43	S
PF00754	F5/8 type C domain	CL0202	L_{-1C}	3JD6$_O$	33	MS
PF00760	Cucumovirus coat protein	CL0055	L_{-1C}	1LAJ$_A$	43	–
PF00775	Dioxygenase	CL0287	L_{-6C}	5VG2$_A$	44	–
PF00777	Glycosyltransferase family 29 (sialyltransferase)	–	L_{+1N}	5BO6$_A$	204	CM

Continued on the next page

Table S3 – continued from previous page

PFAM identifier	PFAM domain	PFAM clan	Lasso types	Representant	Lasso loop size	Cellular location
PF00782	Dual specificity phosphatase, catalytic domain	CL0031	L_{+1N}	$1X24_B$	56	CM
PF00811	Ependymin	–	L_{-1N}; L_{-1N},L_{-1N}; L_{-3N}	$6JL9_A$	98	CS
PF00812	Ephrin	CL0026	L_{-1N}	$2I85_A$	65	M
PF00819	Myotoxin, crotamine	CL0075	L_{-1C}	$1H5O_A$	20	S
PF00840	Glycosyl hydrolase family 7	CL0004	L_{-3N}	$6RWF_A$	126	–
PF00857	Isochorismatase family	–	L_{+1N}	$2WTA_A$	170	–
PF00858	Amiloride-sensitive sodium channel	–	L_{-1N}	$4NTW_B$	22	–
PF00869	Flavivirus glycoprotein, central and dimerisation domains	CL0543	L_{-1C}	$3C5X_C$	35	–
PF00878	Cation-independent mannose-6-phosphate receptor repeat	CL0226	L_{+1C}; L_{+1C},L_{+1C}	$2L21_A$	37	M
PF00879	Defensin propeptide	–	L_{-1C}	$2MXQ_A$	17	S
PF00884	Sulfatase	CL0088	L_{-1N}	$4FDI_A$	112	C
PF00913	Trypanosome variant surface glycoprotein (A-type)	–	L_{-2N}	$6SOY_A$	229	M
PF00915	Calicivirus coat protein	CL0055	L_{-1N}	$5O03_C$	61	CMS
PF00920	Dehydratase family	–	L_{+1N}	$5J83_A$	69	–
PF00925	GTP cyclohydrolase II	–	L_{+1N}	$4RL4_B$	107	C
PF00930	Dipeptidyl peptidase IV (DPP IV) N-terminal region	CL0186	L_{-1C}	$6L8Q_B$	54	CMS
PF00933	Glycosyl hydrolase family 3 N terminal domain	CL0058	L_{-2N}	$3UT0_A$	44	C
PF00944	Alphavirus core protein	CL0124	L_{-1C},L_{-1C}; L_{-1C}	$5VU2_M$	33,34	–
PF00965	Tissue inhibitor of metalloproteinase	CL0353	L_{-2C}	$1D2B_A$	70	MS
PF00967	Barwin family	CL0199	L_{+1C},L_{+1N}	$1BW3_A$	33,58	S
PF00974	Rhabdovirus spike glycoprotein	–	$LL_{-1,+1}$,L_{-1N}; $LL_{+1,-1}$,L_{-1N}; L_{+1C},L_{-1N}	$5OYL_A$	47,48	–
PF00999	Sodium/hydrogen exchanger family	CL0064	LS_{2++C}	$5BZ2_A$	100	M
PF01003	Flavivirus capsid protein C	–	L_{-1C}; L_{-1C},L_{-1C}; $LL_{-1,+1}$,L_{-1C},L_{-1C}; L_{-1N}	$5H37_A$	32	–
PF01015	Ribosomal S3Ae family	–	L_{-2C}	$6SW9_R$	57	C
PF01032	FecCD transport family	CL0142	L_{+1N}	$2QI9_F$	77	M
PF01033	Somatomedin B domain	–	L_{+1C},L_{+2C},L_{-1N},L_{-2C},L_{-2C}; L_{+1C},L_{+2C},L_{-1N},L_{-1C}; L_{+1C},L_{+2C},L_{-1N}	$4B56_A$	388,101, 47,213,84	MS
PF01048	Phosphorylase superfamily	CL0408	L_{+1N}	$1V4N_A$	68	C
PF01082	Copper type II ascorbate-dependent monooxygenase, N-terminal domain	CL0612	L_{-1C}	$1OPM_A$	46	–
PF01083	Cutinase	CL0028	L_{-2C}; L_{+1C}; L_{+1N}	$4PSC_A$	37	S
PF01094	Receptor family ligand binding region	CL0144	L_{+1N}; L_{-1N}; L_{-1C},L_{-1N}; $LL_{+1,-1}$	$1DP4_A$	50	M
PF01108	Tissue factor	CL0159	L_{+1C}	$2HYM_B$	98	MS
PF01109	Granulocyte-macrophage colony-stimulating factor	CL0053	L_{-2N}	$1CSG_A$	34	S
PF01113	Dihydrodipicolinate reductase, N-terminus	CL0063	L_{+1N}	$1B7G_Q$	27	C
PF01120	Alpha-L-fucosidase	CL0058	L_{-1C}	$5K9H_A$	419	C
PF01122	Eukaryotic cobalamin-binding protein	CL0059	L_{+1C}	$2BB5_A$	247	S
PF01129	NAD:arginine ADP-ribosyltransferase	CL0084	L_{+1N}	$1GXY_A$	203	M
PF01156	Inosine-uridine preferring nucleoside hydrolase	–	L_{-1C}	$4I72_A$	106	C
PF01161	Phosphatidylethanolamine-binding protein	–	L_{+1N}	$1FUX_A$	84	M
PF01202	Shikimate kinase	CL0023	L_{+1N}	$2PT5_A$	101	C
PF01244	Membrane dipeptidase (Peptidase family M19)	CL0034	L_{+1N}	$1ITQ_A$	33	M
PF01266	FAD dependent oxidoreductase	CL0063	$LL_{+1,-1}$	$5OC3_A$	55	–
PF01289	Thiol-activated cytolysin	CL0293	L_{+2C}	$5IMY_A$	219	–
PF01291	LIF / OSM family	CL0053	L_{+1C},L_{+1C}	$1A7M_A$	123,114	MS
PF01301	Glycosyl hydrolases family 35	CL0058	L_{-1C}	$5IAZ_A$	54	C
PF01303	Egg lysin (Sperm-lysin)	–	L_{-1N}	$1GAK_A$	75	M
PF01341	Glycosyl hydrolases family 6	–	L_{+1N}	$1DYS_B$	60	S
PF01352	KRAB box	–	L_{+1N}	$4IJD_A$	44	C
PF01356	Alpha amylase inhibitor	–	L_{-1N}	$1HOE_A$	29	–

Continued on the next page

Table S3 – continued from previous page

PFAM identifier	PFAM domain	PFAM clan	Lasso types	Representant	Lasso loop size	Cellular location
PF01390	SEA domain	–	L_{-1N}	$1EAW_B$	22	–
PF01391	Collagen triple helix repeat (20 copies)	–	L_{-1N},L_{-1N}; L_{-1C}	$1LI1_A$	56,59	MS
PF01392	Fz domain	CL0644	L_{-1C}	$5BQC_A$	56	MS
PF01395	PBP/GOBP family	–	L_{+1C}	$2KPH_A$	59	–
PF01400	Astacin (Peptidase family M12A)	CL0126	L_{+1N}	$1AST_A$	157	CS
PF01401	Angiotensin-converting enzyme	CL0126	L_{-2N}; L_{-1C}	$6S1Y_A$	146	–
PF01404	Ephrin receptor ligand binding domain	CL0202	L_{-1N}	$3HEI_B$	61	MS
PF01453	D-mannose binding lectin	CL0186	LS_{3+--C}	$5GYY_A$	39	M
PF01464	Transglycosylase SLT domain	CL0037	L_{+1C}	$1GBS_A$	57	S
PF01471	Putative peptidoglycan binding domain	CL0244	L_{-2C}	$2J0T_E$	70	MS
PF01497	Periplasmic binding protein	CL0043	L_{+1N}; L_{-1N}	$5YSC_A$	81	M
PF01510	N-acetylmuramoyl-L-alanine amidase	–	L_{-2C}	$1YCK_A$	46	–
PF01520	N-acetylmuramoyl-L-alanine amidase	CL0035	L_{+1N}	$4LQ6_A$	49	–
PF01542	Hepatitis C virus core protein	–	L_{-2C},L_{-2C}	$6MEI_C$	75,28	–
PF01543	Hepatitis C virus capsid protein	–	L_{-1N}	$4CL1_C$	49	–
PF01547	Bacterial extracellular solute-binding protein	CL0177	L_{-1N}; L_{+1N}	$4R2B_A$	68	M
PF01562	Reprolysin family propeptide	–	L_{-2C}; L_{-1C}	$3CKI_B$	68	MS
PF01565	FAD binding domain	CL0077	L_{-1C}	$6F74_A$	85	–
PF01567	Hantavirus glycoprotein G1	CL0159	L_{-1C},L_{-1N},L_{-1N},L_{-1N}	$5LJZ_A$	132,125, 36,135	–
PF01570	Flavivirus polyprotein propeptide	–	L_{-1C}	$4UTC_A$	32	–
PF01593	Flavin containing amine oxidoreductase	CL0063	L_{+2C}	$5Z2G_B$	164	S
PF01600	Coronavirus spike glycoprotein S1	–	L_{-1C}	$6B7N_A$	26	–
PF01607	Chitin binding Peritrophin-A domain	CL0155	L_{+1N}	$5ZNS_A$	260	S
PF01611	Filovirus glycoprotein	–	L_{+1N}	$6EA5_A$	27	CM
PF01625	Peptide methionine sulfoxide reductase	–	L_{-1N}	$2IEM_A$	148	C
PF01630	Hyaluronidase	CL0058	L_{+1N}	$2PE4_A$	291	CS
PF01652	Eukaryotic initiation factor 4E	CL0625	L_{-1C}	$2IDR_A$	39	–
PF01657	Salt stress response/antifungal	–	L_{-1C}; L_{-1C},L_{-1C}	$3A2E_A$	77	S
PF01716	Manganese-stabilising protein / photosystem II polypeptide	CL0193	L_{-1C}	$5G38_A$	26	C
PF01742	Clostridial neurotoxin zinc protease	CL0126	L_{-1N}	$6UC6_C$	53	M
PF01764	Lipase (class 3)	CL0028	L_{+1N}	$1USW_A$	230	S
PF01766	Birnavirus VP2 protein	CL0055	L_{-2N}	$3P06_A$	93	–
PF01804	Penicillin amidase	CL0052	L_{-1C}	$4YF9_A$	90	–
PF01823	MAC/Perforin domain	CL0293	L_{-3N}	$3NSJ_A$	167	CMS
PF01826	Trypsin Inhibitor like cysteine rich domain	–	L_{-1N}	$1ATA_A$	39	S
PF01835	MG2 domain	CL0159	L_{-2C}	$4FXG_C$	79	MS
PF01839	FG-GAP repeat	CL0186	L_{+1C},L_{-2C}; L_{+1C}	$3IJE_B$	42,48	M
PF01871	AMMECR1	–	L_{-1N}	$1WSC_A$	57	–
PF01979	Amidohydrolase family	CL0034	L_{+1N}	$3MTW_A$	42	–
PF02014	Reeler domain	CL0159	L_{-1C},L_{-1C},L_{-1C},L_{-1N},L_{-1C}; LS_{3--+C}	$5B4X_C$	40,167, 41,48,41	S
PF02015	Glycosyl hydrolase family 45	CL0199	L_{+1C},L_{+1N},L_{+1N}	$5H4U_A$	104,69,116	S
PF02024	Leptin	CL0053	L_{+1N}	$1AX8_A$	51	S
PF02035	Coagulin	CL0079	L_{+2C},L_{-1C}	$1AOC_A$	86,102	S
PF02059	Interleukin-3	CL0053	L_{+1C}	$2L3O_A$	64	S
PF02087	Nitrophorin	CL0116	L_{-1N}	$1NP1_A$	131	–
PF02098	Tick histamine binding protein	CL0116	L_{-1N}	$1QFT_A$	122	S
PF02140	Galactose binding lectin domain	–	L_{-1C},L_{-1C},L_{-1C},L_{-1C}; L_{-1C},L_{-1C}; L_{-1C}	$5H4S_A$	31,27, 34,32	–
PF02157	Mannose-6-phosphate receptor	CL0226	L_{+1C}	$1C39_A$	36	CM
PF02177	Amyloid A4 N-terminal heparin-binding	–	L_{-1N}; L_{-1C}	$1AAP_A$	22	–
PF02191	Olfactomedin-like domain	CL0434	L_{-1C}	$6NAX_A$	189	CMS
PF02210	Laminin G domain	CL0004	L_{-1N}	$3ASI_A$	29	M

Continued on the next page

Table S3 – continued from previous page

PFAM identifier	PFAM domain	PFAM clan	Lasso types	Representant	Lasso loop size	Cellular location
PF02225	PA domain	CL0364	LS_{2--N}; L_{+1N},L_{-1N},LS_{2--N}; LS_{2--N},L_{+1N},L_{-1N},LS_{2--N}	$1SUV_C$	195	–
PF02244	Carboxypeptidase activation peptide	CL0570	L_{+1C}; L_{-1C},L_{-1C}; L_{-1N},L_{-1N}; L_{-1N},L_{-1N},L_{-1N}	$3HLP_A$	24	–
PF02250	35kD major secreted virus protein	CL0653	L_{+1C},$LL_{+4,-3}$; L_{-2C}; L_{+2C},L_{-1N},L_{-3C}; L_{+1C},$LL_{+4,-2}$; L_{+2C},$LL_{+4,-3}$	$2FFK_A$	189,40	–
PF02265	S1/P1 Nuclease	CL0368	L_{+1C}	$5FB9_A$	145	CS
PF02298	Plastocyanin-like domain	CL0026	L_{-1N}	$1F56_A$	34	CMS
PF02375	jmjN domain	CL0029	LS_{2--N}	$5TVS_B$	73	–
PF02383	SacI homology domain	CL0031	L_{+1C}	$4XUU_A$	66	CM
PF02394	Interleukin-1 propeptide	–	L_{-2C}	$1ITB_B$	44	CMS
PF02404	Stem cell factor	CL0053	L_{-1N}	$2O26_A$	96	CMS
PF02430	Apical membrane antigen 1	CL0168	L_{-1N}	$3ZLE_A$	91	MS
PF02442	Lipid membrane protein of large eukaryotic DNA viruses	–	L_{+2C}	$1YPY_A$	88	–
PF02458	Transferase family	CL0149	L_{-1N}	$2E1T_A$	309	–
PF02469	Fasciclin domain	–	L_{-1C}	$1NYO_A$	135	S
PF02489	Herpesvirus glycoprotein H main domain	–	L_{-2C}	$3PHF_2$	29	–
PF02670	1-deoxy-D-xylulose 5-phosphate reductoisomerase	CL0063	L_{+1N}	$1K5H_A$	193	–
PF02678	Pirin	CL0029	L_{-1C}	$2VEC_A$	195	C
PF02758	PAAD/DAPIN/Pyrin domain	CL0041	L_{+1N}	$4JBM_B$	163	C
PF02784	Pyridoxal-dependent decarboxylase, pyridoxal binding domain	CL0036	L_{-1C}	$5GJO_A$	78	C
PF02798	Glutathione S-transferase, N-terminal domain	CL0172	L_{+1N}	$4OFM_A$	52	–
PF02917	Pertussis toxin, subunit 1	CL0084	L_{-1N}	$4Z9C_A$	152	S
PF02918	Pertussis toxin, subunit 2 and 3, C-terminal domain	CL0658	L_{-1N}	$4K6L_G$	152	S
PF02947	flt3 ligand	CL0053	L_{-1N},L_{-2N}	$1ETE_A$	84,40	MS
PF03045	DAN domain	CL0079	L_{-1C}	$5AEJ_A$	51	S
PF03067	Lytic polysaccharide mono-oxygenase, cellulose-degrading	CL0159	L_{-1C}	$4OPB_A$	102	M
PF03098	Animal haem peroxidase	CL0617	L_{-3C}	$2E9E_A$	162	–
PF03122	Herpes virus major capsid protein	–	L_{+1N}	$6ODM_5$	132	–
PF03146	Agrin NtA domain	CL0353	L_{-2C}; L_{-1N}	$1JC7_A$	73	MS
PF03150	Di-haem cytochrome c peroxidase	CL0318	L_{-2C},L_{-1C}	$3L4M_C$	33,32	–
PF03165	MH1 domain	CL0263	L_{-1C}	$1MHD_A$	46	C
PF03167	Uracil DNA glycosylase superfamily	–	L_{+1N}	$3ZOQ_A$	119	–
PF03254	Xyloglucan fucosyltransferase	–	L_{+1N},L_{-1C}	$5KOR_B$	128,28	CM
PF03330	Lytic transglycolase	CL0199	L_{+1C},L_{+1N}; L_{+1C}	$2HCZ_X$	29,68	MS
PF03401	Tripartite tricarboxylate transporter family receptor	CL0177	L_{+1N}; $LL_{+1,-1}$	$2F5X_A$	37	M
PF03480	Bacterial extracellular solute-binding protein, family 7	CL0177	LS_{2--N}	$3FXB_A$	142	M
PF03489	Saposin-like type B, region 2	CL0707	L_{-2N}	$5W7E_B$	331	CS
PF03523	Macrophage scavenger receptor	–	L_{-1C}	$6J02_A$	65	M
PF03583	Secretory lipase	CL0028	L_{+1N}	$2VEO_A$	173	S
PF03921	Intercellular adhesion molecule (ICAM), N-terminal domain	CL0011	L_{+1N}	$1MQ8_B$	139	–
PF03958	Bacterial type II/III secretion system short domain	–	L_{+1N}	$6HCG_P$	55	M
PF03968	LptA/(LptD N-terminal domain) LPS transport protein	CL0259	L_{+1C},L_{+1C}; L_{+3C}	$5IV9_A$	690,549	M
PF03973	Triabin	CL0116	L_{-1N}	$4N7C_A$	132	–
PF03996	Hemagglutinin esterase	CL0264	L_{-1C}; L_{-1C},L_{-1C}	$5JIF_A$	50	–
PF04092	SRS domain	–	L_{-1N}	$5WA2_A$	122	M
PF04137	Endoplasmic Reticulum Oxidoreductin 1 (ERO1)	–	$LL_{-1,+1}$	$1RP4_A$	146	CM
PF04143	Sulphur transport	–	L_{+1C}	$6LEO_A$	70	M
PF04170	NlpE N-terminal domain	CL0116	L_{+1N}	$2Z4H_B$	67	M
PF04185	Phosphoesterase family	CL0088	$LL_{+1,+1}$	$2D1G_A$	54	–

Continued on the next page

Table S3 – continued from previous page

PFAM identifier	PFAM domain	PFAM clan	Lasso types	Representant	Lasso loop size	Cellular location
PF04430	Protein of unknown function (DUF498/DUF598)	–	L_{+1C}	$1IHN_A$	83	–
PF04454	Encapsulating protein for peroxidase	CL0373	L_{+1C}	$6I9G_A$	121	S
PF04734	Neutral/alkaline non-lysosomal ceramidase, N-terminal	–	L_{-1C}	$2ZXC_A$	49	S
PF05096	Glutamine cyclotransferase	CL0186	L_{-1N}	$2FAW_A$	79	–
PF05108	Type VII secretion system ESX-1, transport TM domain B	–	L_{-1C}	$6SGY_A$	200	M
PF05222	Alanine dehydrogenase/PNT, N-terminal domain	CL0325	L_{+1N}	$2Q99_A$	45	–
PF05337	Macrophage colony stimulating factor-1 (CSF-1)	CL0053	L_{-1N},L_{-2N}	$3EJJ_A$	92,45	MS
PF05388	Carboxypeptidase Y pro-peptide	–	L_{+1N}	$1CPY_A$	243	C
PF05431	Insecticidal Crystal Toxin, P42	–	$LL_{-1,-1}$	$5FOY_B$	95	S
PF05463	Sclerostin (SOST)	CL0079	L_{-1C}	$2KD3_A$	55	S
PF05550	Pestivirus Npro endopeptidase C53	–	L_{-1N}	$1S4F_B$	52	–
PF05609	Lamina-associated polypeptide 1C (LAP1C)	CL0023	L_{+1N}	$4TVS_B$	159	CM
PF05637	galactosyl transferase GMA12/MNN10 family	CL0110	L_{+1N}	$6BSU_A$	223	CM
PF05922	Peptidase inhibitor I9	CL0570	L_{+1N}	$4DZT_A$	33	–
PF06083	Interleukin-17	CL0079	LS_{2--C}	$4QHU_C$	97	S
PF06119	Nidogen-like	–	L_{-1N}	$1NPE_A$	219	MS
PF06296	RelE toxin of RelE / RelB toxin-antitoxin system	CL0136	L_{-1N}	$5JA8_B$	53	CMS
PF06309	Torsin	CL0023	L_{+1N}	$5J1T_B$	159	CM
PF06328	Ig-like C2-type domain	CL0159	L_{+1C},L_{+1C}	$1PVH_B$	123,114	MS
PF06416	Effector protein NleG	CL0229	L_{-1N}	$2KKY_A$	37	–
PF06433	Methylamine dehydrogenase heavy chain (MADH)	CL0186	L_{-2C},L_{-1C}	$3C75_M$	33,32	M
PF06446	Hepcidin	–	L_{-3N}	$4QAE_A$	100	–
PF06484	Teneurin Intracellular Region	–	L_{-1C}	$6SKE_B$	31	CM
PF06511	Type III secretion systems tip complex components	–	L_{-1N}	$5VXK_B$	59	CMS
PF06652	Methuselah N-terminus	–	L_{-1C}	$1FJR_A$	95	M
PF06702	Golgi casein kinase, C-terminal, Fam20	CL0016	L_{-1C},L_{-1C}	$5WRR_A$	111,113	CS
PF06744	Type VI secretion protein IcmF C2-like domain	CL0154	L_{-1N}	$4Y7M_A$	57	CMS
PF07137	VDE lipocalin domain	CL0116	L_{-1N}	$3CQR_A$	132	CM
PF07243	Phlebovirus glycoprotein G1	–	L_{-1C}	$5Y10_C$	148	M
PF07246	Phlebovirus nonstructural protein NS-M	–	L_{-1N},L_{-1C}; L_{-1C}; L_{-3N},L_{-1C}	$4HJ1_A$	195,49	–
PF07249	Cerato-platanin	CL0199	L_{+1C},L_{+1N}	$3SUJ_A$	38,61	S
PF07502	MANEC domain	CL0168	LS_{3+--C}; L_{-1N}	$2MSX_A$	43	S
PF07519	Tannase and feruloyl esterase	CL0028	L_{+1C}	$6FAT_A$	52	S
PF07648	Kazal-type serine protease inhibitor domain	CL0005	L_{-1N}	$3V65_A$	27	MS
PF07653	Variant SH3 domain	CL0010	L_{-1N}	$1HJD_A$	72	S
PF07654	Immunoglobulin C1-set domain	CL0011	L_{-1C}	$2QEJ_A$	60	–
PF07679	Immunoglobulin I-set domain	CL0011	L_{-1C},L_{-1C}; L_{-1C}; L_{+1C},L_{-2C}; L_{+1C}	$5FTT_C$	31,183	M
PF07686	Immunoglobulin V-set domain	CL0011	L_{-2C}; L_{-1N}; L_{+1C}; L_{+1N}; L_{-3N}; L_{-1C}; L_{-1C},L_{-1C}	$5CBA_E$	28	MS
PF07691	PA14 domain	CL0301	LS_{2--C},LS_{2--N}	$4GQ7_A$	120,88	M
PF07714	Protein tyrosine and serine/threonine kinase	CL0016	L_{-1C}	$5GZA_A$	68	CM
PF07715	TonB-dependent Receptor Plug Domain	–	L_{+1N},L_{-1N},LS_{2--N}; LS_{3--+N},L_{+1N},L_{-1N},LS_{2--N}	$3V89_B$	192,273, 220	–
PF07728	AAA domain (dynein-related subfamily)	CL0023	L_{-1C}	$6P0F_A$	54	C
PF07732	Multicopper oxidase	CL0026	L_{-2N}; L_{-2N},L_{+1N}; L_{-1N}	$5LDU_A$	404	–
PF07745	Glycosyl hydrolase family 53	CL0058	L_{+1N}	$1FHL_A$	59	MS
PF07883	Cupin domain	CL0029	L_{-1C}	$3BU7_A$	224	C
PF07936	Potassium-channel blocking toxin	CL0075	L_{-1C}	$1BDS_A$	27	S
PF07966	A1 Propeptide	–	L_{-1N}; $LL_{-1,+1}$	$1LYA_A$	70	CS
PF07974	EGF-like domain	CL0001	L_{-1C},L_{+1N},L_{-1N}	$4Z80_A$	203,99,73	M

Continued on the next page

Table S3 – continued from previous page

PFAM identifier	PFAM domain	PFAM clan	Lasso types	Representant	Lasso loop size	Cellular location
PF07992	Pyridine nucleotide-disulphide oxidoreductase	CL0063	L_{+2N}; L_{+1C}; L_{+1N}	2YAU$_A$	125	–
PF08127	Peptidase family C1 propeptide	–	L_{-1N}	1CPJ$_A$	67	CMS
PF08131	Defensin-like peptide family	CL0075	L_{-1C}	1D6B$_A$	17	S
PF08189	Meleagrin/Cygnin family	CL0075	L_{-1C}	2MJK$_A$	17	S
PF08205	CD80-like C2-set immunoglobulin domain	CL0011	L_{+1N}	5VKJ$_A$	129	M
PF08212	Lipocalin-like domain	CL0116	L_{-1N}	5EZ2$_A$	125	S
PF08246	Cathepsin propeptide inhibitor domain (I29)	–	L_{-1N}	5A24$_A$	54	–
PF08282	haloacid dehalogenase-like hydrolase	CL0137	L_{+1N}	1NF2$_A$	231	C
PF08448	PAS fold	CL0183	L_{-1C}	6EIB$_A$	67	–
PF08534	Redoxin	CL0172	L_{+1N}; L_{-1C}; L_{-1N}	1OC3$_C$	105	C
PF08617	Kinase binding protein CGI-121	–	LS_{2--N}	1ZD0$_A$	84	–
PF08702	Fibrinogen alpha/beta chain family	–	L_{+1C}	1FZA$_B$	86	–
PF08773	Cathepsin C exclusion domain	–	L_{-1N}	1K3B$_A$	83	C
PF08798	CRISPR associated protein	CL0362	$LL_{+1,-1}$	5CD4$_I$	111	CS
PF08924	Domain of unknown function (DUF1906)	CL0058	L_{+1N}	1SFS$_A$	180	–
PF09022	Staphostatin A	CL0354	L_{+1N}	1OH1$_A$	40	C
PF09117	MiAMP1	CL0333	L_{-1N},L_{-1C}	1C01$_A$	54,27	S
PF09207	Yeast killer toxin	CL0333	L_{-1N},L_{-1C}	1WKT$_A$	62,32	S
PF09258	Glycosyl transferase family 64 domain	CL0110	L_{+2N}	1ON8$_A$	53	CM
PF09286	Pro-kumamolisin, activation domain	CL0570	L_{-1C}	3EDY$_A$	162	C
PF09394	Chagasin family peptidase inhibitor I42	CL0159	L_{-1N}	3E1Z$_B$	48	–
PF09691	Type II secretion system pilotin lipoprotein (PulS$_O$utS)	–	L_{+1N}	4A56$_A$	55	C
PF09992	Phosphodiester glycosidase	–	L_{-1C}	6U11$_A$	34	–
PF10182	Flo11 domain	CL0321	L_{+1C}	5FV5$_A$	131	M
PF10250	GDP-fucose protein O-fucosyltransferase	CL0113	L_{+1N}	3ZY3$_B$	88	C
PF10282	Lactonase, 7-bladed beta-propeller	CL0186	L_{-1N}	3U4Y$_A$	272	–
PF10468	Carboxypeptidase inhibitor I68	CL0075	L_{-1C},L_{-1C}; L_{-1C}	2JTO$_A$	18,18	S
PF10528	GLEYA domain	CL0301	LS_{2--C},LS_{2--N}; LS_{2--C}; LS_{2--C},$LL_{-1,+1}$,LS_{2--N}	5A3L$_A$	115,88	M
PF10564	Sialic-acid binding micronemal adhesive repeat	–	LS_{3+--N},LS_{3+--N}	2JH1$_A$	37,46	C
PF10613	Ligated ion channel L-glutamate- and glycine-binding site	CL0177	L_{-1N}	4YKI$_B$	56	CM
PF11032	ApoM domain	CL0116	L_{-3N}	2WEW$_A$	89	S
PF11475	Virion protein N terminal domain	–	L_{-1C}	4NZ0$_A$	112	–
PF12032	Regulatory CLIP domain of proteinases	CL0678	L_{-1C}	2IKD$_A$	32	S
PF12308	Neurogenesis glycoprotein	–	L_{-1C}	6QM3$_A$	183	CMS
PF12708	Pectate lyase superfamily protein	CL0268	L_{+1C}; L_{+3C}; L_{-1N},L_{-1N}	3EQN$_A$	420	–
PF12727	PBP superfamily domain	CL0177	L_{-1N}	4JWO$_A$	110	–
PF12804	MobA-like NTP transferase domain	CL0110	L_{+1N}	3NGW$_A$	169	C
PF12849	PBP superfamily domain	CL0177	L_{+1N}	4OMB$_A$	45	MS
PF12947	EGF domain	CL0001	L_{-1N}	6ZYA$_A$	56	CMS
PF12999	Glucosidase II beta subunit-like	–	L_{+1C}	2LVX$_A$	30	C
PF13091	PLD-like domain	CL0479	L_{+1C}	2ZE4$_A$	47	S
PF13343	Bacterial extracellular solute-binding protein	CL0177	L_{-1N}	4R72$_A$	97	–
PF13399	LytR cell envelope-related transcriptional attenuator	–	L_{+1N}	6Q10$_A$	59	M
PF13407	Periplasmic binding protein domain	CL0144	L_{+1N}	2IPM$_A$	224	M
PF13456	Reverse transcriptase-like	CL0219	L_{+3N}	2EHG$_A$	88	C
PF13458	Periplasmic binding protein	CL0144	L_{+1N}	4N03$_A$	259	–
PF13472	GDSL-like Lipase/Acylhydrolase family	CL0264	L_{-1C}; L_{+1N}	5B5L$_A$	32	S
PF13531	Bacterial extracellular solute-binding protein	CL0177	L_{-1C}	3AXF$_A$	143	M
PF13627	Prokaryotic lipoprotein-attachment site	CL0421	L_{-1N}	1S4I$_A$	94	M
PF13640	2OG-Fe(II) oxygenase superfamily	CL0029	L_{-1N}	3GZE$_A$	36	C

Continued on the next page

Table S3 – continued from previous page

PFAM identifier	PFAM domain	PFAM clan	Lasso types	Representant	Lasso loop size	Cellular location
PF13653	Glycerophosphoryl diester phosphodiesterase family	CL0384	L_{+1N}	$4Q6X_A$	145	S
PF13841	Beta defensin	CL0075	L_{-1C}	$5KI9_A$	15	S
PF13908	Wnt and FGF inhibitory regulator	–	L_{-1N}	$5M0W_A$	19	CM
PF13987	YedD-like protein	–	L_{-2N}	$4HWM_A$	57	–
PF14200	Ricin-type beta-trefoil lectin domain-like	CL0066	L_{-1N}	$3HZB_A$	62	–
PF14416	PMR5 N terminal Domain	–	L_{-1C}	$6CCI_A$	297	CM
PF14497	Glutathione S-transferase, C-terminal domain	CL0497	L_{+1N}	$2WB9_A$	171	–
PF14537	Cytochrome c3	CL0317	$LL_{+2,-1}$	$1Q9I_A$	180	M
PF14565	Interleukin 22 IL-10-related T-cell-derived-inducible factor	CL0053	L_{+1C}	$1M4R_A$	93	S
PF14845	beta-acetyl hexosaminidase like	CL0546	L_{-1N}	$3NSM_A$	20	C
PF14862	Big defensin	CL0075	L_{-1C}	$6QBK_A$	19	S
PF14865	Macin	CL0054	L_{-1C}	$2K35_A$	44	MS
PF15177	Interleukin-28A	CL0053	L_{+1C}	$3HHC_B$	100	S
PF15447	N-terminal segments of PfEMP1	–	L_{-1N}	$2YK0_A$	81	M
PF15508	beta subunit of N-acylethanolamine-hydrolyzing acid amidase	–	L_{-3C}	$5U81_A$	310	C
PF15902	Sortilin, neurotensin receptor 3,	CL0434	L_{-3C}	$5NMT_B$	471	CM
PF16010	Cytochrome domain of cellobiose dehydrogenase	CL0559	L_{+6N}	$4QI7_A$	45	CS
PF16358	RcsF lipoprotein	CL0522	L_{-1C}	$2Y1B_A$	45	M
PF16414	Niemann-Pick C1 N terminus	CL0644	L_{+1N}	$5JNX_C$	27	CM
PF16470	Peptidase S8 pro-domain	CL0570	$LL_{+1,-1}$	$4OMC_A$	150	CMS
PF16499	Alpha galactosidase A	CL0058	L_{+1N}; L_{+1N},L_{-1N}; L_{-1N}	$3A21_A$	41	C
PF16649	Interleukin 23 subunit alpha	CL0053	L_{-1N}	$4GRW_F$	53	CMS
PF16656	Purple acid Phosphatase, N-terminal domain	CL0159	L_{+1N}	$6GIT_A$	82	–
PF16822	SGNH hydrolase-like domain, acetyltransferase AlgX	CL0264	L_{-1C}	$4KNC_B$	186	M
PF16841	Ca-dependent carbohydrate-binding module xylan-binding	CL0202	L_{-1N}	$2XFD_A$	12	–
PF17484	N-Lobe handle Tf-binding protein B	–	LS_{3--+N},L_{+1N},L_{-1N},LS_{2--N}	$3VE1_B$	195,273, 220,192	CMS
PF17808	Fn3-like domain from Purple Acid Phosphatase	CL0159	L_{+2N}	$3ZK4_A$	165	–
PF17858	Platypus intermediate defensin-like peptide	CL0075	L_{-1C}	$2MN3_A$	15	S
PF17859	Pelovaterin	CL0075	L_{-1C}	$2JR3_A$	17	S
PF17860	RK-1-like defensin	CL0075	L_{-1C}	$1EWS_A$	15	S
PF17900	Peptidase M1 N-terminal domain	CL0672	L_{-1C}	$6ATK_E$	28	M
PF17967	Pullulanase N2 domain	–	L_{+1N}	$4CVW_C$	56	C
PF17996	Carbohydrate esterase 2 N-terminal	CL0202	L_{-1C}	$4XVH_A$	173	–
PF18158	Adaptive response protein AidB N-terminal domain	CL0544	L_{-1N}	$3DJL_A$	513	C
PF18193	Fibrillin 1 unique N-terminal domain	CL0001	L_{-1N}	$1UZJ_A$	26	C
PF18207	Leukemia inhibitory factor receptor N-terminal domain	CL0159	L_{+1C},L_{+1C}	$2Q7N_B$	123,114	MS
PF18338	Lower baseplate protein N-terminal domain	CL0606	L_{-1N}	$5E7F_A$	67	CMS
PF18426	Tle cognate immunity protein 4 C-terminal domain	–	L_{-1C}	$5XMG_A$	194	–
PF18452	Immunoglobulin domain	CL0011	L_{-2C}; L_{+1C},L_{-2C}	$1G0Y_R$	44	CMS
PF18487	Thrombospondin type 1 repeat	–	L_{-2N},L_{-2C}; L_{-1N}	$6RUR_B$	641,73	S
PF18611	IL-3 receptor alpha chain N-terminal domain	CL0159	L_{+1C}	$5UV8_B$	69	MS
PF18626	Glutaminase	CL0125	L_{-1N},L_{-1N}; L_{-1N},L_{-1N},$LL_{+1,+2}$	$2KSV_A$	97,50	–
PF18911	PKD domain	CL0159	$LL_{+1,-1}$	$6NZ0_A$	73	–
PF19429	Evasins Class A	–	L_{-2C}	$3FPU_B$	25	S

References

[Arolas et al., 2012] Arolas, J. L., Broder, C., Jefferson, T., Guevara, T., Sterchi, E. E., Bode, W., Stöcker, W., Becker-Pauly, C. and Gomis-Rüth, F. X. (2012). Structural basis for the sheddase function of human meprin β metalloproteinase at the plasma membrane. Proceedings of the National Academy of Sciences *109*, 16131–16136.

[Dabrowski-Tumanski et al., 2016] Dabrowski-Tumanski, P., Niemyska, W., Pasznik, P. and Sulkowska, J. I. (2016). LassoProt: server to analyze biopolymers with lassos. Nucleic Acid Research .

[Niemyska et al., 2016] Niemyska, W., Dabrowski-Tumanski, P., Kadlof, M., Haglund, E., Sułkowski, P. and Sulkowska, J. I. (2016). Complex lasso: new entangled motifs in proteins. Scientific reports *6*, 36895.

[Thangudu et al., 2008] Thangudu, R. R., Manoharan, M., Srinivasan, N., Cadet, F., Sowdhamini, R. and Offmann, B. (2008). Analysis on conservation of disulphide bonds and their structural features in homologous protein domain families. BMC structural biology *8*, 1.

[Tsai et al., 1999] Tsai, J., Taylor, R., Chothia, C. and Gerstein, M. (1999). The packing density in proteins: standard radii and volumes. Journal of molecular biology *290*, 253–266.
